# Molecular recognition at the opioid-modulating neuropeptide FF receptor 1

**DOI:** 10.1093/procel/pwaf090

**Published:** 2025-11-05

**Authors:** Man Na, Yang Yue, Kexin Xu, Ziyi Xu, Lu Zhang, Fangfang Zhou, Jolien De Neve, Steven Ballet, Fei Xu

**Affiliations:** iHuman Institute, ShanghaiTech University, Shanghai 201210, China; School of Life Science and Technology, ShanghaiTech University, Shanghai 201210, China; iHuman Institute, ShanghaiTech University, Shanghai 201210, China; iHuman Institute, ShanghaiTech University, Shanghai 201210, China; School of Life Science and Technology, ShanghaiTech University, Shanghai 201210, China; iHuman Institute, ShanghaiTech University, Shanghai 201210, China; School of Life Science and Technology, ShanghaiTech University, Shanghai 201210, China; iHuman Institute, ShanghaiTech University, Shanghai 201210, China; iHuman Institute, ShanghaiTech University, Shanghai 201210, China; Research Group of Organic Chemistry, Departments of Chemistry and Bioengineering Sciences, Vrije Universiteit Brussel, Pleinlaan 2, B-1050, Brussels, Belgium; Research Group of Organic Chemistry, Departments of Chemistry and Bioengineering Sciences, Vrije Universiteit Brussel, Pleinlaan 2, B-1050, Brussels, Belgium; iHuman Institute, ShanghaiTech University, Shanghai 201210, China; School of Life Science and Technology, ShanghaiTech University, Shanghai 201210, China; Shanghai Academy of Natural Sciences (SANS), Shanghai 200032, China

**Dear Editor,**

The NPFFR1 (neuropeptide FF receptor 1) responds to endogenous RF-amide peptides like NPFF (Neuropeptide FF) and RFRP-3 (RF-amide related peptide-3), both containing a conserved C-terminal Arg-Phe-NH_2_ motif (Quillet et al., 2016). In mammals, NPFF-related peptides are derived from two precursors (Bonini et al., 2000): pro-NPFF_A_, yielding peptides such as NPFF and NPAF (Neuropeptide AF) that prefer binding to NPFFR2 (neuropeptide FF receptor 2); and pro-NPFF_B_, yielding RFRP-3, which favors NPFFR1 (Quillet et al., 2016). NPFFR1 primarily couples to inhibitory Gi/o proteins and regulates diverse physiological functions, including energy homeostasis, reproduction, substance abuse disorders, cardiovascular control, anxiety, food intake, and pain (Nguyen et al., 2020).

Previous studies have suggested that NPFF receptors and their endogenous ligands are involved in the regulation of pain perception and opioid-induced antinociception (Ayachi and Simonin, 2014). The two receptors (NPFFR1 and NPFFR2) may exert opposing pain modulation effects, which may be attributed to the functional differences between NPFFR1 and NPFFR2. While NPFFR1 may act as an anti-opioid modulator, NPFFR2 may instead exert antinociceptive and opioid-potentiating effects (Malin et al., 2015). Blocking NPFFR1 alone can prevent analgesic tolerance and dependence, enhance opioid antinociceptive effects, and reduce opioid ­withdrawal symptoms. However, due to the lack of highly selective agonists and antagonists of NPFFR1 vs NPFFR2, their precise mechanism of opioid modulation is yet to be unraveled.

RFRP-3 and NPFF are endogenous ligands of NPFFR1, both sharing the four-amino acid sequence PQRF at their C-termini (Fig. 1A and 1B). However, the remaining four amino acids at the N-termini of each peptide result in different potencies at NPFFR1. This was confirmed through GloSensor assay (to measure the cAMP levels) using 293 T cells expressing NPFFR1 to assess the potency of these two ligands (Fig. 1C). While both ligands activate the receptor’s Gi signaling pathway, RFRP-3 exhibit ∼20-fold increase of potency to NPFFR1 compared to NPFF (EC_50_ = 1.99 nmol/L and 39.8 nmol/L for RFRP-3 and NPFF, respectively; Table S1). To investigate the underlying mechanisms of ligand recognition (the structural differences for the two RF-amide peptides bound to NPFFR1) and subtype-selectivity (between NPFFR1 and NPFFR2), we carried out structural studies for the two ligand-bound NPFFR1-Gi complexes. We designed the NPFFR1 construct as described in the Methods section. Cryo-EM maps were acquired at 3.16 Å and 3 Å global resolution for the NPFF-NPFFR1-Gi complex and RFRP-3-NPFFR1-Gi complex, respectively (Figs. 1D, 1E, S2 and S3; Table S2), and the atomic models were built and refined according to the cryo-EM maps. Local refinement of the receptor region yielded maps at 4.02 Å and 3.77 Å resolution for the NPFF-bound and RFRP-3-bound NPFFR1 structures, respectively.

**Figure 1.**
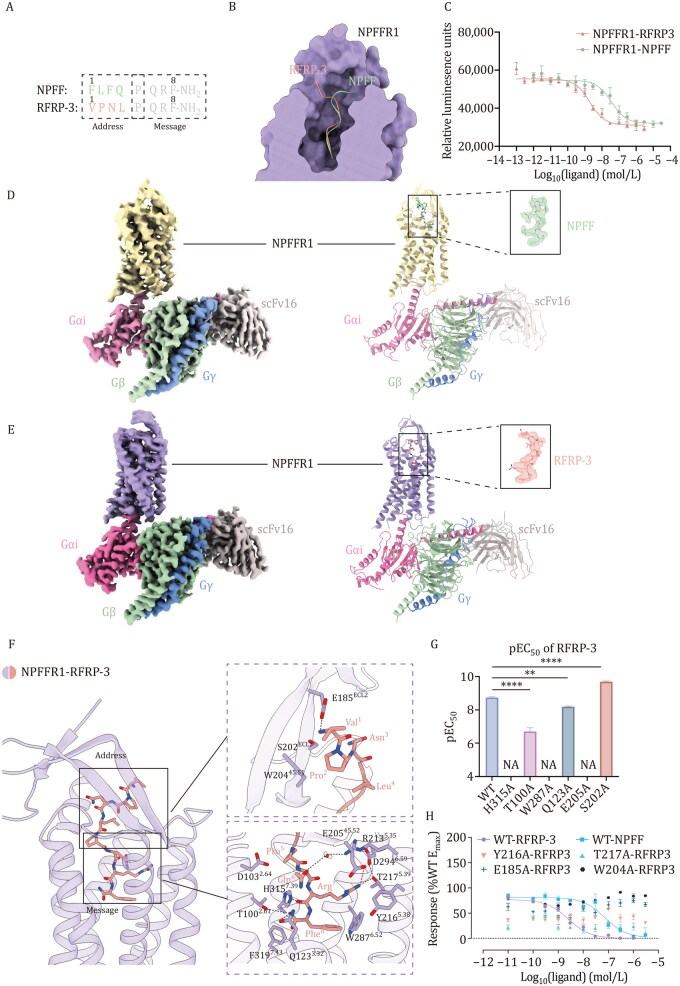
**Cryo-EM structures of NPFF-NPFFR1-Gi and RFRP-3-NPFFR1-Gi complexes and ligand recognition mechanism**. (A) The ligand sequences of NPFF and RFRP-3 are presented, with the distinct sequences of the two peptides’ N-termini emphasized as colored “address” parts, and the identical C-termini indicated as “message” parts. The residue numbering from the N-termini to the C-termini is shown above the sequence in black. (B) Differences in the binding poses of NPFF and RFRP-3 to NPFFR1 within the orthosteric pocket. (C) The cAMP accumulation assay for NPFFR1 activation upon NPFF and RFRP-3 binding. Data shown are mean ± S.E.M. of three independent experiments (*n* = 3). (D and E) Cryo-EM maps and structure models of NPFF-NPFFR1-Gi complex (D) and RFRP-3-NPFFR1-Gi (E) complex. NPFF, light green; RFRP-3, light orange; NPFFR1, yellow (NPFF-bound) or purple (RFRP-3-bound); Gα_i_, Gβ, and Gγ are in pink, green, and blue, respectively. scFv16 is shown in grey. (F) Key residues of NPFFR1 when bound to RFRP-3. The residues involved in recognition are colored in purple. Hydrogen bonds are shown in grey dashed lines, ionic bonds are shown in red dashed lines. (G) Effects of key residue mutations on Gi signaling in the RFRP-3 binding pocket of NPFFR1 are represented by pEC50, measured by the GloSensor cAMP accumulation assay. Each column represents the data of an independent replicate (*n *≥ 3). The corresponding data are shown in Table S1. “NA” indicates no detectable signal. Statistical significance was assessed using one-way ANOVA. (H) Dose–response curves of NPFFR1 mutants. Data shown are mean ± S.E.M. of three independent experiments (*n* = 3).

We first analyzed the ligand binding mode for the two peptides. RFRP-3 and NPFF bind similarly to the bottom of NPFFR1’s orthosteric pocket. Their C-terminal PQRF motifs insert into the core binding site formed by TM2/3, TM5/6, and TM7, while the N-termini interact with ECL2 (Extracellular loop 2) (Fig. 1D and 1E). Such a two-segment binding mechanism aligns with the “message-address” concept of different peptide hormones, including opioid ligands binding to the opioid receptors (Fig. S5), where the message part is crucial for recognition and activation, and the address part is crucial for subtype selectivity (Lee et al., 2015). Here, we designate the N-terminal segment for the two RF-amid peptides as “address” part and the C-terminal segment as “message” part. The primary ­difference between the two structures is found in the orientations of the N-terminal address segment, attributable to sequence variation. The N-terminus of RFRP-3 leans toward TM3 and TM4, whereas NPFF leans toward TM1 (Figs. 1B, 1D, and 1E and S6).

The C-terminal QRF-NH_2_ segment of NPFF and RFRP-3 mediates key interactions with NPFFR1, consistent with the previous findings that the C-terminal residues in the RF-amide peptide family are crucial for receptor recognition and activation (Vyas et al., 2006), conferring the “message” function. Both three-amino acid peptide segments adopt nearly identical poses, with only subtle differences in the Gln6 side chain (Figs. 1F and  S7). Of note, Phe^8^ residue forms a T-shaped π–π interaction with W287^6.52^ (Figs. 1F and S7). The α-amide of Phe^8^ is stabilized by three residues: T100^2.61^, Q123^3.32^ and H315^7.39^ through hydrogen bonds. The side chains of Arg^7^ and E205^45.52^ form a salt-bridge interaction (Figs. 1F, S7 and S9). The key roles of these residues are supported by mutagenesis and cAMP accumulation assays (Fig. 1G and S8; Table S1). Additionally, D294^6.59^ may participate in the hydrogen bond network formed by T217^5.39^ and Arg^7^ of RFRP-3 (Fig. 1F and 1H), supported by a previous study showing that D^6.59^ A mutation results in a significant loss in ligand affinity and receptor activation (Findeisen et al., 2011). Although NPFF and RFRP-3 share an identical *C*-terminal PQRF motif, our structural comparison revealed that the detailed interaction networks at this region are not fully shared. In the RFRP-3-bound structure, Arg7 engages in a more extensive hydrogen bonding network involving D294^6.59^ and T217^5.39^, which appears less stable in the NPFF-bound complex. This structural divergence may contribute to the higher potency of RFRP-3 toward NPFFR1 relative to NPFF.

In contrast to the message segments, the address parts of the ligands, located at their N-termini, display major differences in their binding mode, as can be observed in the two structures. RFRP-3’s N-terminus leans toward ECL2 and forms hydrogen bonds and hydrophobic interactions with E185^ECL2^ and W204^45.51^; alanine mutations at these sites abolished activation (Fig. 1F and 1H; Table S1). In contrast, NPFF’s N-terminus forms fewer contacts and appears more flexible (Figs. S7 and S9), contributing to its lower potency (Fig. 1C). Our structural analysis supports previous findings showing that truncating the N-terminal three amino acids (FLF) of NPFF reduces its potency by 2-fold for NPFFR1 activation and 8-fold for NPFFR2 activation (Findeisen et al., 2011), whereas truncating the four N-terminal amino acids (VPNL) of RFRP-3 led to a greater loss of potency toward NPFFR1 (Rouméas et al., 2015). Although NPFF and RFRP-3 showed nearly identical binding affinities for NPFFR1 in previous radioligand assays (Rouméas et al., 2015), our structural data reveal that RFRP-3 forms additional stabilizing contacts in the N-terminal “address” region, suggesting that the ∼20-fold potency enhancement of RFRP-3 may be attributed to the enhanced receptor conformational stability during ­activation and G protein coupling efficiency.

It has been reported that RFRP-3 and NPFF exhibit different selectivity toward NPFFR1 and NPFFR2, despite the sequence similarity between the two receptors being as high as 50% (Bonini et al., 2000). Sequence alignment reveals high similarity in TM regions but marked differences in ECL2 and ICL3 (Intracellular loop 3) (Fig. S10). To elucidate the mechanisms by which the endogenous ligands, NPFF specifically, interact differently with NPFFR1 and NPFFR2, we aligned our structure with the recently reported hNPSF-bound NPFFR2 structure (Kim et al., 2025). Of note, hNPSF has three additional residues at the N-terminus (H-SQAFLFQPQRF-NH2), as compared to NPFF (Fig. S11). The ligands, NPFF and hNPSF, adopt similar conformations in their message regions, located within the conserved binding pockets of both receptors.

In contrast to the overall similarity in the binding pockets for the message part, the address regions of the ligands exhibit distinct interactions with the extracellular residues of the receptors (Fig. S11). Moreover, since hNPSF contains three additional amino acids compared to NPFF, its binding pose in NPFFR2 could be different from that of NPFF. To eliminate the effect introduced by the different peptides, we used AlphaFold3 to predict NPFF–NPFFR2 model and identified three ECL2 residues that interact with the ligand N-terminus: Y190^ECL2^, W205^ECL2^, and R207^45.51^ (Fig. 2A). In NPFFR1, W204^45.51^ replaces R207^45.51^ (Fig. 2A), likely disrupting a hydrogen bond with NPFF seen in NPFFR2, resulting in the reduced stability of ECL2. Although the overall resolution of ECL2 is limited due to its intrinsic flexibility, the density corresponding to residue 45.51 is clearly defined (Fig. S12). We therefore hypothesize that residue at position 45.51 (ECL2) contributes to NPFF’s preference for NPFFR2 over NPFFR1. Consistently, mutation of W204^45.51^ in NPFFR1 to arginine led to a significant increase in NPFF-induced Gi-signaling activation efficacy, whereas the reciprocal mutation R207^45.51^W in NPFFR2 caused a decrease in efficacy, as demonstrated by the cAMP accumulation assay (Fig. S13; Tables S1 and S3). MD simulation analysis further confirmed higher conformational stability of the receptor, the ECL2 region and the ligand in the NPFFR1^W204R^ mutant over 300 ns simulation compared to the WT (Figs. 2B, 2C and S14), supporting the functional importance of position 45.51 in conferring subtype selectivity.

**Figure 2.**
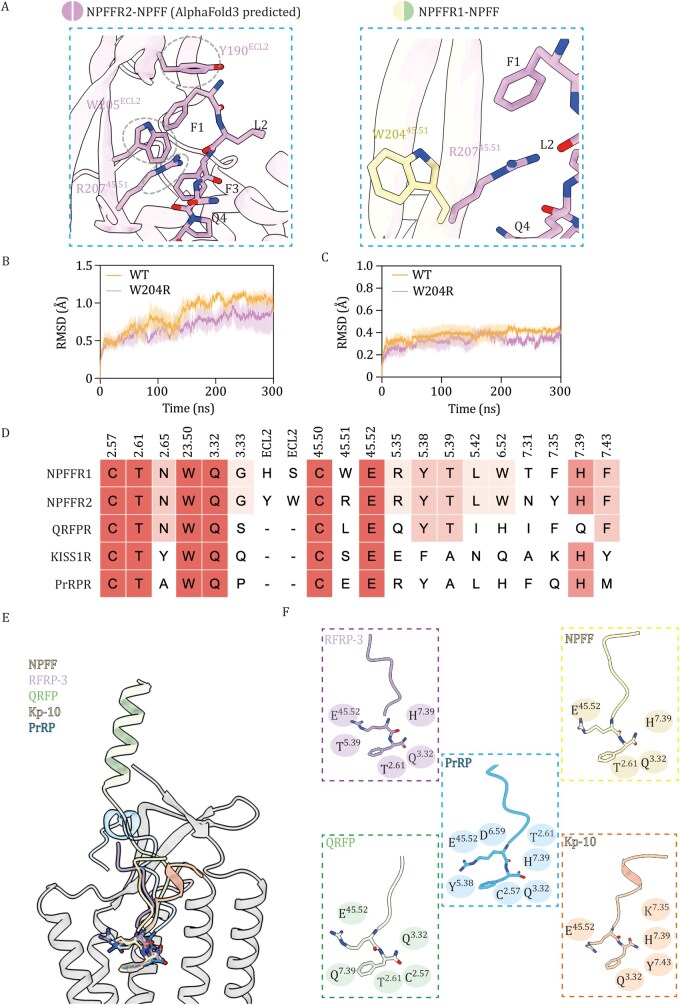
**Subtype selectivity and recognition pattern analysis of the RF-amide peptides**. (A) AlphaFold3 predicted binding mode of NPFF to NPFFR2; ECL2 residues involved in molecular interactions are highlighted by grey dashed circles. NPFFR1 and NPFFR2 are aligned together to show the residue difference. (B and C) Molecular dynamics simulations were conducted for 300 ns to compare the root mean square deviation of the protein complex (B) and the bound ligand NPFF (C) between the wild-type NPFFR1 (WT) and the W204^45.51^R mutant, which corresponds to the equivalent residue in NPFFR2 (*n *= 3). (D) Sequence alignment of the orthosteric binding pockets across RF-amide family receptors, with conserved residues highlighted in color (dark red for absolute conservation; light red for relative conservation). (E) Structural comparison of RF-amide peptides’ C-termini (depicted as colored sticks) bound to receptor (represented by NPFFR1), including QRFP (PDB: 8WZ2), Kp-10 ( PDB: 8ZJD), PrRP ( PDB: 8ZPS), RFRP-3 and NPFF. (F) Key interactions between RF-amide peptides and their receptors, including hydrogen bonds and ionic bonds, are labeled around the ligands.

Next, to understand the recognition mechanism of the RF-amide peptide family, we compared binding pockets across several receptor structures, including QRFPR (Pyroglutamylated RF-amide peptide receptor) (PDB: 8WZ2), KISS1R (Kisspeptin Receptor) (PDB: 8ZJD) and PrRPR (Prolactin-releasing peptide receptor) (PDB: 8ZPS) (Li et al., 2024; Jin et al., 2024; Shen et al., 2024). Conserved residues at positions 2.57, 2.61, 23.50, 3.32, 45.50, 45.52, and 7.39 mediate key interactions with the C-terminal Arg–Phe motifs of RF-amide peptides (Fig. 2D–F). Despite the general resemblance in the binding conformations of RF-amide family peptides (Fig. 2E and 2F), subtle differences in receptor sequences lead to variations in ligand binding modes in the “message” region. Remarkably, residue T^5.39^ of NPFFR1 and NPFFR2 establishes an essential hydrogen bond with the Arg residue of their endogenous ligands, as its mutation in both NPFFR1 and NPFFR2 abolished the ligand-induced signaling (Fig. 1H; Tables S1 and S3). This is consistent with the interactions observed in the NPY-NPY1R and PP-NPY4R structures (Tang et al., 2022). However, such interactions were not observed in other RF-amide ligand-receptor structures (Fig. 2F). Notably, NPFFR1/2 feature three negatively charged regions in their binding pockets (Fig. S15), which may complement the positively charged motifs found in all five RF-amide ­peptide families and underscore their broader ligand ­recognition landscape (Quillet et al., 2016). Furthermore, structural comparison with the AlphaFold-predicted ­inactive-state NPFFR1 structure implies that NPFFR1 activation involves the canonical conformational changes typical of class A GPCRs required for G protein coupling (Fig. S16).

Future efforts in the design of selective NPFFR1 ligands could benefit from structural insights presented in this study, such as (i) elongating the ligand’s N-terminus to enable more extensive and specific interactions with the receptor’s extracellular loops, as was done in case of N-terminal elongation-NPFF (SQA/SPA/NPA—NPFF) in past work for selectivity towards NPFFR2 (Mollereau et al., 2002). Additionally, to enhance NPFFR1 selectivity, replacing the peptide N-terminal Val with polar residues (e.g., Ser, Thr, Asn, Asp, Cys, Pen or Dap) may promote hydrogen bonding with S202 in NPFFR1, while the corresponding W202 in NPFFR2 would not provide a suitable binding partner and may even introduce steric clashes when inserting bulky residues into the peptide ligand; (ii) modifying the fourth amino acid (Leu4) could promote selective interactions with the extracellular residue R213^5.35^ in NPFFR1, which has a distinct orientation towards the peptide ligand could therefore enable subtype selectivity; and (iii) imposing conformational constraints on the peptide ligands to favor either interactions with NPFFR1 or NPFFR2. In addition, the current data will also facilitate the design of bifunctional or even multitarget ligands that target NPFFRs and other receptors, as was done previously by us and others during the development of opioid-NPFFR ligands. Making use of the structural data provided in this work, the N-terminal part of such ligands or the linker part between two active pharmacophores could steer NPFFR selectivity.

Collectively, our research offers significant insights into the structural basis for ligand recognition, selectivity, and activation mechanism of NPFFR1, which can provide crucial guidance for the development of highly selective pharmacological tools and drug design targeting NPFFR1 for the benefits of the treatment of opioid-associated disorders.

## Footnotes

We thank Junlin Liu, Suwen Hu, Na Chen, Qiwen Tan, Qiaoyun Shi, Lin Wang, Xiaoyan Liu, Pei Si for protein cloning, expression and assay support; Sen Zheng, Li Wang, Dandan Liu, Qianqian Sun, and Yuan Pei at the Bio-EM facility at ShanghaiTech University for technical support on cryo-EM data collection.

This work was supported by the Bilateral Research Foundation Flanders (FWO-Vlaanderen)—National Science Foundation China (NSFC) (Grant Nos. W2412030 and G0AO725N), and Innovative Research Team of high-level local universities in Shanghai. F.X. is a SANS Exploration Scholar. J.D.N. and S.B. acknowledge the Research Council of the Vrije Universititeit Brussel for financial support through the Strategic Research Programme 95 (SRP95).

The authors declare no conflict of interest. The authors declare their agreement to participate. The authors declare their agreement to publish. This paper does not report original code.

The Cryo-EM density maps and the atomic model coordinates for the NPFF-NPFFR1-Gi complex and RFRP-3-NPFFR1-Gi complex have been deposited in the Electron Microscopy Data Bank with accession code EMD-65089 and EMD-65081, and the Protein Data Bank with accession code 9VIF and 9VI9, respectively. The local maps of the receptor region of NPFF-bound and RFRP-3-bound NPFFR1 have been deposited with accession codes EMD-66288 and EMD-66853, respectively.

All authors reviewed and approved the article. M.N. performed cloning and purification of NPFFR1-Gi complexes, performed cryo-EM sample preparation, data collection, structure analysis and MD simulation; M.N. and Y.Y. performed cryo-EM data processing and structure determination; K.X. and Z.X. designed and optimized GloSensor assays; F.Z. and L.Z. performed GloSensor assays, M.N. and Y.Y. prepared the related figures; J.D.N. and S.B. participated in the interpretation of the data; F.X. conceived and supervised the project. M.N. Y.Y., J.D.N., S.B. and F.X. wrote the manuscript.

This article used AlphaFold3 to predict binding mode of NPFF to NPFFR2.

## Supplementary Material

pwaf090_Supplementary_Data
