# Supplementary material for: Molecular recognition at the opioid-modulating neuropeptide FF receptor 1
Source: Protein Cell. 2025 Nov 5;17(4):372–7. doi: 10.1093/procel/pwaf090 (PMC13107563; doi:10.1093/procel/pwaf090)
Supplement: pwaf090_Supplementary_Data [file pwaf090_supplementary_data.docx]

**Supplemental materials for**

**Title:** **Molecular Recognition at the Opioid-modulating Neuropeptide FF Receptor 1**

**Authors:**

Man Na^1,2,#^, Yang Yue^1,#,*^, Kexin Xu^1,2^, Ziyi Xu^1,2^, Lu Zhang^1^, Fangfang Zhou^1^, Jolien De Neve^3^, Steven Ballet^3^, Fei Xu^1,2,*^

**Affiliations:**

^1^iHuman Institute, ShanghaiTech University, Shanghai, China

^2^School of Life Science and Technology, ShanghaiTech University, Shanghai, China

^3^Research Group of Organic Chemistry, Departments of Chemistry and Bioengineering Sciences, Vrije Universiteit Brussel, Pleinlaan 2, B-1050 Brussels, Belgium

^#^These authors contributed equally to this work.

^*^Corresponding authors:

Yang Yue: yueyang@shanghaitech.edu.cn; Fei Xu: xufei@shanghaitech.edu.cn

The supplemental materials include:

- Materials and methods of this paper
- Figure S1-S16
- Table S1-S3
- References for the supplemental materials

# **Materials and methods**

**Cloning and co-expression of NPFFR1 and Gi heterotrimer for cryo-EM study**

The human WT NPFFR1, encoded by NPFFR1 gene, was cloned into the pFastBac1 vector, excluding 60 residues from the C-terminus, and introduced a F^3.41^W thermostable mutation to improve protein expression. The C-terminal fusion MBP, and an N-terminal fusion, including a prolactin precursor sequence as a signal peptide (Zhuang, Wang et al. 2022), Flag tag and BRIL fusion (Chun, Thompson et al. 2012), were added. Human Gαi1, with three dominant-negative alterations (S47N, G203A, A326S) (Liang, Zhao et al. 2018) and Gβ1γ2, was independently cloned into the pFastBac1 and pFastBacDual vectors. For generating NPFFR1-Gi complex, human NPFFR1, Gαi1, Gβ1, and Gγ2 were co-expressed in Trichuplusia ni Hi5 insect cells by the Bac-to-bac operation system. Cells got infected by baculoviruses for NPFFR1, Gαi1, and Gβ1γ2 in a proportion of 1:3:2 at 2×10^6^ cells for each mL. Regarding NPFFR1's individual expression, solely the NPFFR1 baculovirus was strained to inoculate the cells. After 48 hours of growing them at 27 °C following infection, cells were accumulated via centrifugation and deposited at -80 °C.

**Expression and purification of scFv16**

Utilizing methods previously published (Hua, Li et al. 2020), we expressed and purified scFv16, a single-chain antibody fragment with an 8x His tag, from Trichuplusia ni Hi5 insect cells. We sub-cloned scFv16 into the pFastBac1 vector and subsequently expressed this protein as a secretory protein in insect cells. We initially isolated the protein using Ni-NTA affinity chromatography. This involved modifying the supernatant's pH to 8.0 with 1M Tris and allowing it to combine with Ni-NTA resin at 4°C for 2 hours. We loaded the resin into a gravity column and rinsed it sequentially with 6 column volumes (CV) of wash buffer I (containing 20 mM HEPES at pH 7.5, 100 mM NaCl, and 10 mM imidazole) and 4CV of wash buffer II (containing 20 mM HEPES at pH 7.5, 100 mM NaCl, and 30 mM imidazole). Lastly, we removed scFv16 from the resin using an Elute buffer (containing 20 mM HEPES with pH 7.5, 100 mM NaCl, and 250 mM imidazole).

**Purification and formation of NPFFR1-Gi-scFv16 complex**

We utilized a previously reported method to express and purify NPFFR1-Gi co-expressed culture from 1L of cell pellets. We resuspended the cell pellets in 120 mL of hypotonic buffer, which contained 10 mM HEPES (pH 7.5), 10 mM MgCl2, 20 mM KCl, and a protease inhibitor cocktail (Roche). The suspension was incubated at 4 °C overnight after adding 10 µM RFRP-3/NPFF (GenScript, Nanjing, China) and 0.25 U Apyrase (Sigma, 0.5 U/µL). Following a centrifugation (140,000 x g) step for 20 minutes at 4 °C, we resuspended the resulting pellet in 18 mL of the same hypotonic buffer. We achieved solubilization by mixing this resuspended pellet with an equal volume of solubilization buffer – composed of 100 mM HEPES (pH 7.5), 200 mM NaCl, 1% (w/v) LMNG (Anatrace), and 0.2% (w/v) CHS (Sigma) supplemented with 10 µM RFRP-3 or NPFF and 0.08 U Apyrase – followed by a 2-hour incubation at 4 °C. The solubilized supernatant was collected after a similar centrifugation step. Following this, we added 50 µL of Talon superflow metal affinity resin (Clontech) and 20 µM imidazole to the solubilized supernatant and allowed it to incubate overnight at 4 °C. We loaded this mixture onto an Econo-Pac disposable chromatography column and washed with 14 CV of wash buffer. The buffer comprised 50 mM HEPES (pH 7.5), 100 mM NaCl, 5% glycerol, 0.01% (w/v) LMNG, 0.002% (w/v) CHS, 30 mM imidazole, and 10 µM RFRP-3/NPFF. Subsequently, we eluted the complex with 4 CV of elution buffer, containing 50 mM HEPES (pH 7.5), 100 mM NaCl, 5% glycerol, 0.01% (w/v) LMNG, 0.002% (w/v) CHS, 200 mM imidazole, and 10 µM RFRP-3/NPFF. After combining the eluted protein with 125 µg of scFv16 and incubating it for another hour at 4 °C, the mixture was subjected to size-exclusion chromatography using a pre-equilibrated Superdex 200 10/300 GL column (GE Healthcare). The eluates were then concentrated to a final protein concentration of 1 mg/mL after SDS-PAGE analysis.

**Cryo-EM sample preparation and data collection**

We treated a Holey carbon grid (CryoMatrix Amorphous alloy film R1.2/1.3, 300 mesh) with glow-discharge for 45 seconds in a H_2_/O_2_ environment. Afterwards, we applied 3 μL of the complex onto the grid. To prepare the sample, we used a Vitrobot Mark IV (Thermo Fisher Scientific, USA), setting the chamber to 100% humidity and 4 °C, with the sample preparation parameters at a blot time of 3 seconds and blot force of -1. Subsequently, we collected the cryo-EM dataset on a Titan Krios 300kV electron microscope (Thermo Fisher Scientifics, USA). The magnification, already calibrated, was set at 165,000, providing a pixel size of 0.832 Å/pixel for NPFF-NPFFR1-Gi, RFRP-3-NPFFR1-Gi complex. We made each movie from 40 frames with a total dose of 60 e^-^/Å^2^, and set the dose rate at 15e^-^/Å^2^/s. For data collection, we used SerialEM v3.8.0 software and provided a defocus range of -0.7 μm to -2.2 μm.

**Cryo-EM image processing and 3D reconstruction**

Two datasets of total 11,340 movies were used to produce the density map of NPFF-NPFFR1-Gi complex via cryoSPARC v3.0 software (Punjani, Rubinstein et al. 2017). The patch motion correction algorithm was used to correct the beam-induced motion artifacts. Followed is the determination of the Contrast Transfer Function (CTF) parameters for each dose-weighted micrograph using the patch CTF estimation module. A total of 4,279,887 and 2,835,194 particles were picked and extracted in each dataset using the Auto blob picking algorithm. These particles then underwent two or three rounds of 2D classification to generate template with clearer complex information. The templates were then input into template picking, followed by one or two rounds of 2D classification. Initial models were reconstructed using the selection of particles. These models were used for further heterogeneous refinement rounds to conduct 3D classification. As a result, three clusters of particles were classified, among which 127,984 and 142,054 particles were combined for further 3D reconstruction. The subsets were subjected to final non-uniform refinement and yielded a density map of 3.16 Å for the NPFF-NPFFR1 complex, and 4.03 Å at the receptor region. The resolution was generated based on the gold standard Fourier shell correlation (FSC) at the 0.143 threshold. Local resolution was determined using the local resolution estimation tool within cryoSPARC. DeepEMhancer was utilized to improve the local density and to sharpen the density map (Sanchez-Garcia, Gomez-Blanco et al. 2021).

For the structural analysis of the RFRP-3-NPFFR1-Gi complex, two datasets of movies comprising of 7,054 movies and 2,252 movies were captured and processed using cryoSPARC v3.0 software (Punjani, Rubinstein et al. 2017). The patch motion correction algorithm was used to correct the beam-induced motion artifacts. Followed is the determination of the Contrast Transfer Function (CTF) parameters for each dose-weighted micrograph using the patch CTF estimation module. A total of 3,373,531 particles and 1,381,953 particles were picked and extracted in each dataset using the Auto blob picking algorithm. These particles then underwent two or three rounds of 2D classification to generate template with clearer complex information. The templates were then input into template picking, followed by three rounds of 2D classification. Initial models were reconstructed using the selection of particles. These models were used for further heterogeneous refinement round to conduct 3D classification. As a result, three clusters of particles were classified, among which 140,756 particles were combined for further 3D reconstruction. The subsets were subjected to final non-uniform refinement and yielded a density map of 3 Å for the RFRP-3-NPFFR1 complex, and 3.77 Å for the receptor region. The resolution was generated based on the gold standard Fourier shell correlation (FSC) at the 0.143 threshold. Local resolution was determined using the local resolution estimation tool within cryoSPARC. DeepEMhancer was utilized to improve the local density and to sharpen the density map (Sanchez-Garcia, Gomez-Blanco et al. 2021).

**Cryo-EM model building and refinement**

Models with PDB ID  7W0M (Yue, Liu et al. 2022) served as reference for constructing and refining the target structures using electron density data. Initial model placement within the cryo-EM density map was performed in UCSF Chimera v1.15 (Pettersen, Goddard et al. 2004), followed by manual adjustments and iterative rebuilding in Coot v0.8.9 (Emsley, Lohkamp et al. 2010). Real-space refinement was conducted using Phenix v1.14 (McCoy, Grosse-Kunstleve et al. 2007). Model validation and quality assessment were carried out with MolProbity 4.2 (Chen, Arendall et al. 2010). Structural visualizations and figure preparation were completed using UCSF Chimera, ChimeraX v1.2.4, and PyMOL v2.5.1 (<http://www.pymol.org>). Detailed refinement statistics are provided in Table S2. The receptor's canonical seven-transmembrane helices, as well as its extracellular and intracellular loops, were mostly well-defined (Fig. S4). However, the N-terminal residues M1-T28, the third intracellular loop A246-P259, and the C-terminal residues G339-R370 were not resolved as clearly as the rest of the receptor. The side chain of Phe^3^ and Phe^1^of NPFF has been removed due to unresolved density.

**GloSensor cAMP accumulation assay**

To conduct cAMP accumulation assay, WT (wild-type) and mutant NPFFR1 gene was cloned into the pcDNA3.1 vector, with a fusion of an N-terminal hemagglutinin (HA) signal sequence and a Flag epitope. The HEK293T cells (ATCC, CRL-11268) stably expressing NPFFR1 were propagated in Dulbecco's Modified Eagle Medium (1x DMEM, Life Technologies) enriched with 1% dialyzed fetal bovine serum (dFBS) and maintained in a controlled atmosphere of 5% CO_2_ at 37 °C. To assess the impact of QuickChange PCR-derived mutations on the NPFFR1-Gi protein signaling pathway, a split luciferase GloSensor cAMP biosensor assay (Promega) was employed. 24 hours before conducting the assay, HEK293T cells were co-transfected with 1 μg of NPFFR1 DNA alongside 1 μg of pGloSensor™-22FGloSensor cAMP DNA (Promega) using Lipofectamine 2000 (Life Technologies) in 6 cm dish via Calcium phosphate precipitation method. Following a 24-hour culture period, cells were seeded into poly-L-lysine-coated 384-well white assay plates (Greiner) at a density of 10,000-15,000 cells within 40 μL of medium per well and incubated overnight (16-20 hours). Subsequent experiments commenced with the addition of 20 μL of 2 mg/mL D-luciferin sodium salt solution in Hanks’ Balanced Salt Solution (HBSS, pH 7.4) to each well, followed by a 1-hour incubation at 37 °C. Agonist-induced and constitutive activities were evaluated by introducing 10 μL of agonists in buffer to the wells, reaching final concentrations in the range of 0 to 30 μM, and incubating for 15 minutes at 37 °C. For the Gi agonist assay, wells received an additional 10 μL of isoproterenol (Sigma) to achieve a final concentration of 200 nM, followed by a 2015-minute incubation at 37 °C. The intracellular cAMP levels were quantified using an EnVision multi-plate reader (Perkin Elmer) and data were processed with GraphPad Prism software version 9.0. Cell surface expression of wild-type and mutant NPFFR1 was quantified using fluorescence-activated cell sorting (FACS). Briefly, cells expressing NPFFR1 were incubated with an anti-Flag M2–fluorescein isothiocyanate (FITC)-conjugated antibody (Sigma) for 20 minutes at 4 °C. The cells were subsequently washed with HBSS, and the surface expression levels of NPFFR1 were determined by measuring the FITC fluorescence intensity via a Guava EasyCyte HT flow cytometer (Millipore).

**Molecular dynamics simulation of NPFFR1**

The initial PDB model used in molecular dynamics simulation underwent several preparations in Maestro software, including the deletion of the scFv16 component, adding of the missing intracellular loop 3 (ICL3) using the protein linker design, and adding the mutation of W3.41F to retrieve the wild type state of the receptor. The protein complex was prepared in the Schrödinger software (Sastry, Adzhigirey et al. 2013), including hydrogen addition, terminal capping. The residue D2.50 was protonated to mirror the protonation state that take place during GPCR activation (Ranganathan, Dror et al. 2014). The prepared structures of the NPFF-NPFFR1WT-Gi and NPFF-NPFFR1W204R-Gi were further processed in the CHARMM-GUI platform membrane builder (Wu, Cheng et al. 2014). OPM database was used to orient the receptor in the membrane bilayer (Lomize, Pogozheva et al. 2012). The models were embedded into a lipid bilayer composed of 386 POPC molecules and solvated into a TI3P water box containing 0.15 M NaCl, forming a simulation box of 120*120*200.

For molecular dynamics simulation under the Gromacs v.2023.3 software, CHARMM36m force filed was selected (Vanommeslaeghe and MacKerell 2012), and CGenFF tool was used to generate the force field of ligand NPFF (Huang and MacKerell 2013, Huang, Rauscher et al. 2017). The systems underwent an initial energy minimization process, followed by a 250 ps of NVT ensemble that gradually heats the system to 310 K, using a time step of 1 fs. NPT ensemble was then carried out under 310 K and 1 atm semi-isotropic condition for 31.25 ns, gradually reducing positional restraints in protein, ligand and lipid atoms. Post-equilibration, pre-production simulation was conducted to generate repeats independently. The production MD was conducted for 300 ns with an integration time step of 2 fs. The LINCS algorithm (Hess 2008) was used to place constraints on bonds involving hydrogen atoms. Electrostatic interactions were calculated using the particle mesh Ewald method with a 12 Å cutoff (Darden, York et al. 1993). Trajectory analysis was performed on GROMACS, with a timestep of 50 ps.

**Data availability**

The Cryo-EM density maps and the atomic model coordinates for the NPFF-NPFFR1-Gi complex and RFRP-3-NPFFR1-Gi complex have been deposited in the Electron Microscopy Data Bank with accession code EMD-65089 and EMD-65081, and the Protein Data Bank with accession code 9VIF and 9VI9, respectively.

**Supplementary figures**


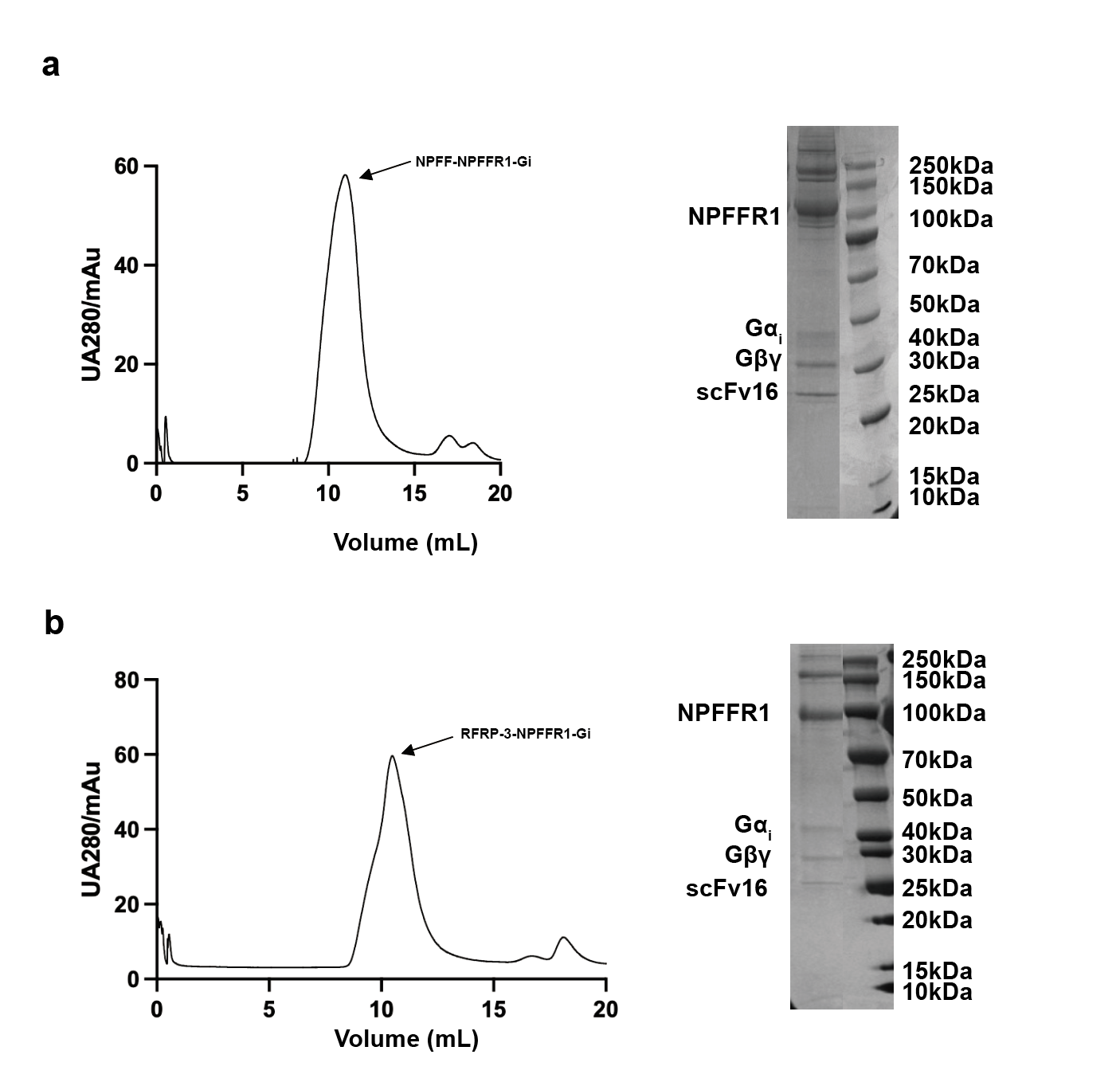
**Fig. S1. The expression and purification of NPFFR1-Gi complex. a-b.** Size-exclusion chromatography (SEC) profile and SDS-PAGE gel result of the NPFF-NPFFR1-Gi **(a)** and RFRP-3-NPFFR1-Gi **(b)** complexes.


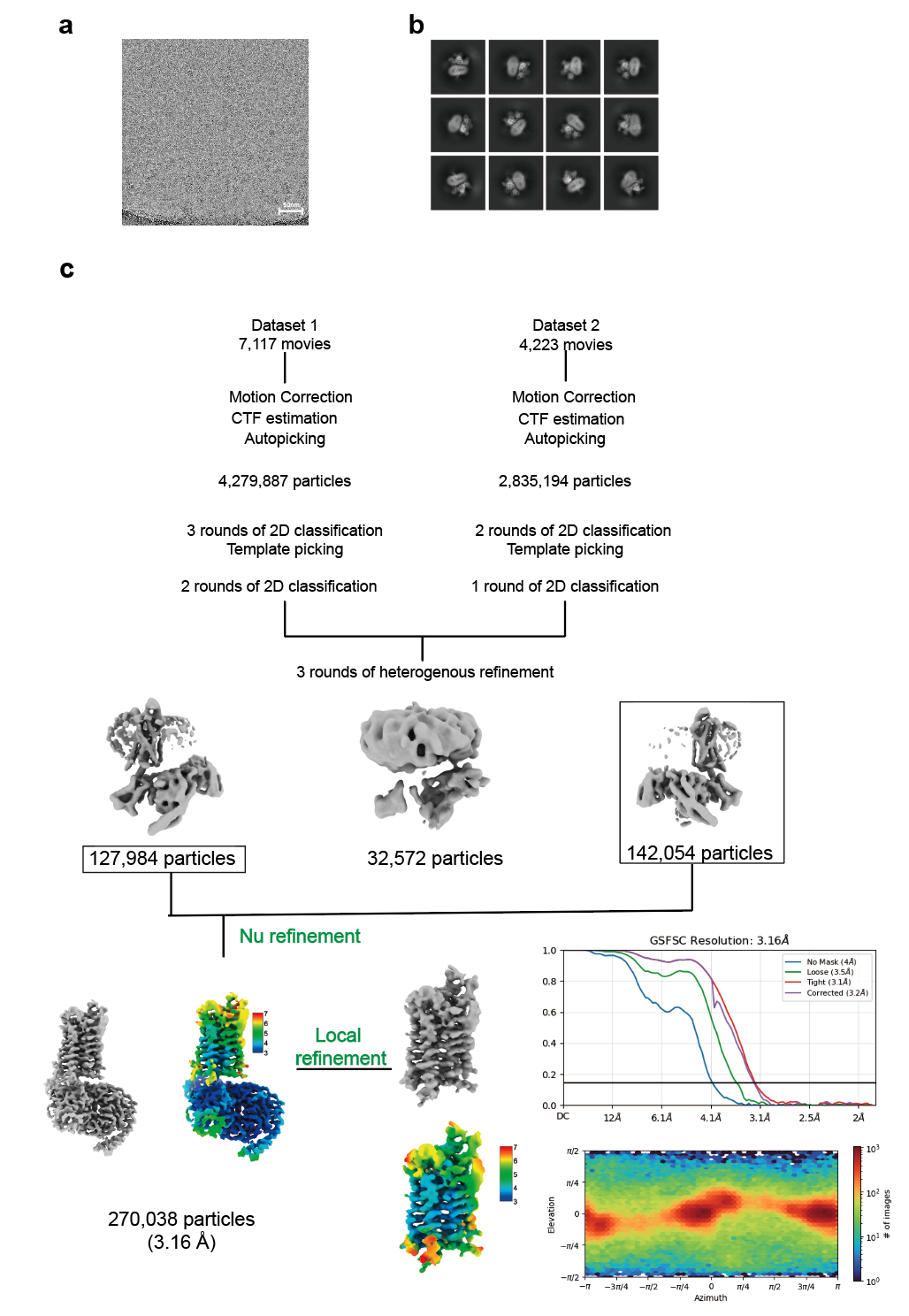


**Fig. S2. Cryo-EM data processing of NPFF-NPFFR1-Gi complex. a.** Representative cryo-EM image of the dataset, with a scale bar of 50 nm. **b**. Representative 2D averages of NPFF-NPFFR1-Gi complex. **c.** Cryo-EM data processing workflow with maps colored by local resolution (Å).


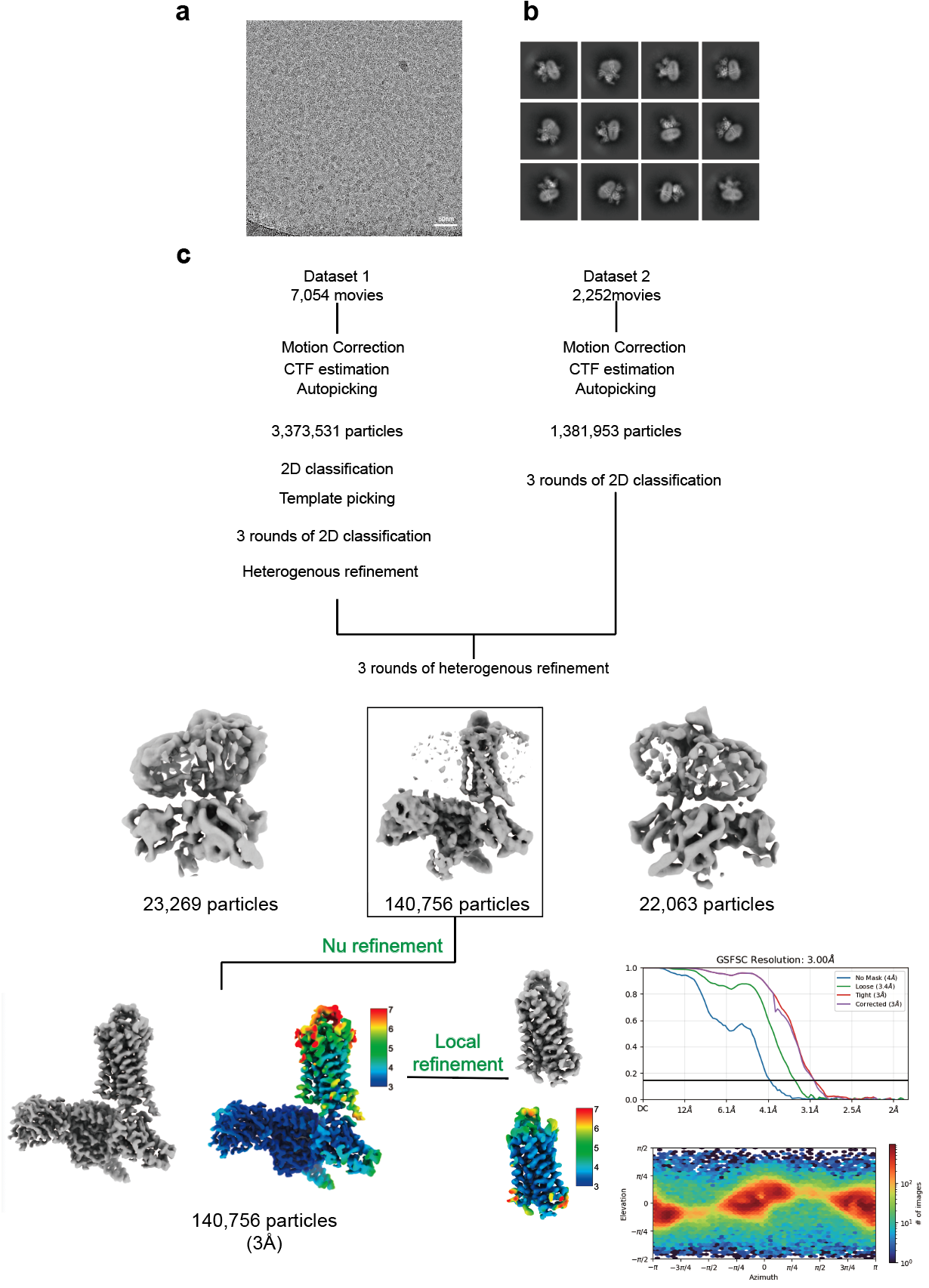


**Fig. S3. Cryo-EM data processing of RFRP-3-NPFFR1-Gi complex. a.** Representative cryo-EM image of the dataset, with a scale bar of 50 nm. **b**. Representative 2D averages of RFRP-3-NPFFR1-Gi complex. **c.** Cryo-EM data processing workflow with maps colored by local resolution (Å).


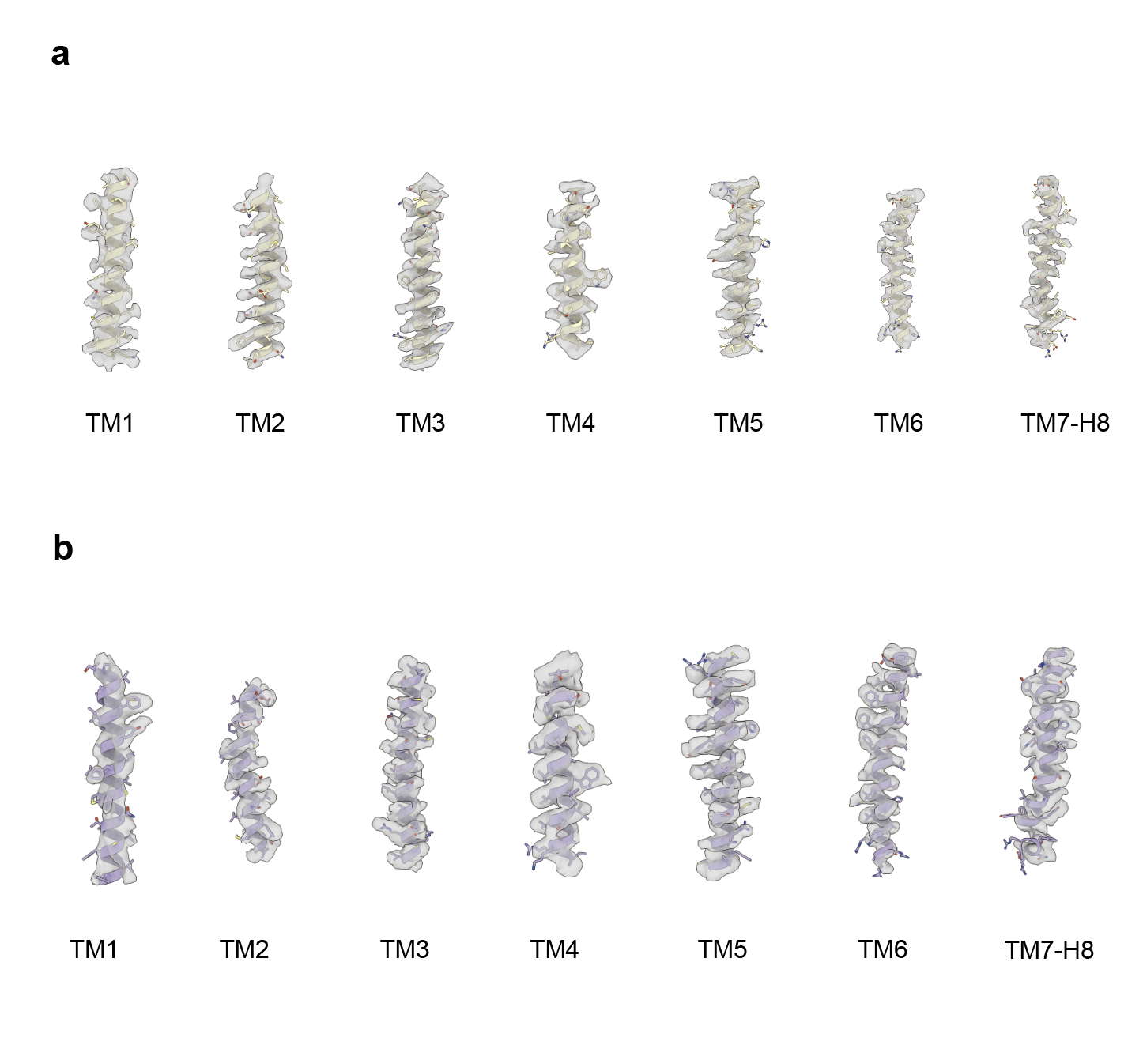


**Fig. S4. Cryo-EM density maps and models of representative helices from the NPFF/RFRP-3-NPFFR1-Gi complexes. a.** Cryo-EM maps and models for NPFF- NPFFR1-Gi. **b.** Cryo-EM maps and models for RFRP-3-NPFFR1-Gi.


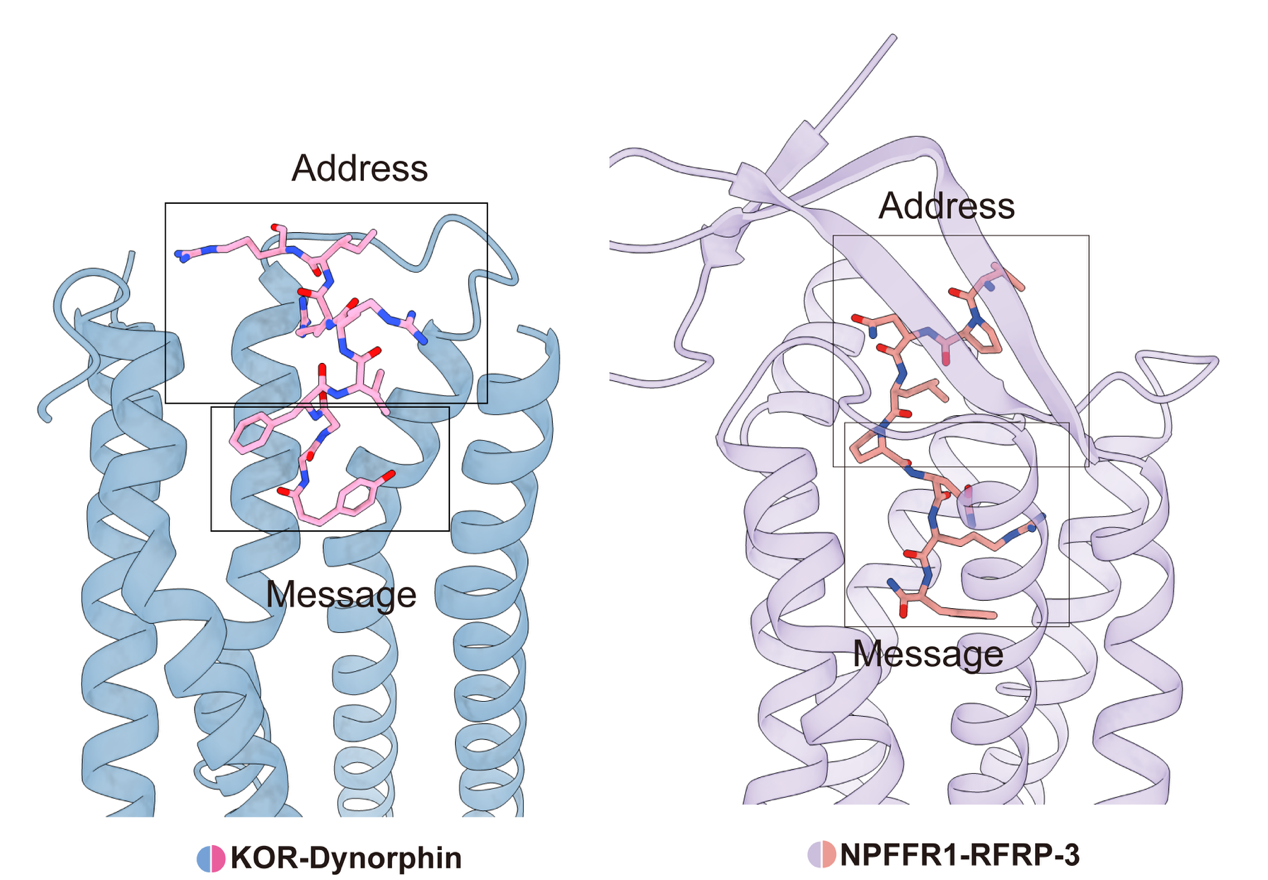


**Fig. S5. Comparison of the “message-address” between κ-opioid receptor (KOR)-dynorphin-A (1–13) (PDB: 7Y1F) and NPFFR1-RPRF-3 complexes.** KOR is shown in blue, dynorphin-A (1–13) in pink; NPFFR1 is depicted in purple, RPRF-3 in light orange.


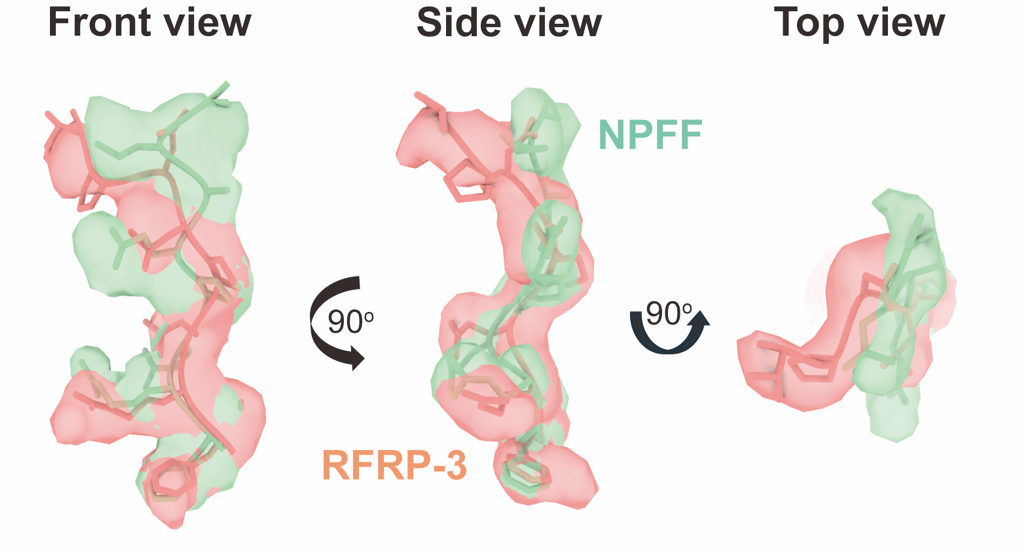


**Fig. S6. Comparison of ligand conformations in two complex structures.** The peptides are overlaid by cryo-EM density map for NPFF-bound and RFRP-3-bound structures, respectively. RFRP-3 is shown in light orange, NPFF is shown in light green, side chains of Phe 1 and Phe 3 have been deleted due to unresolved density.


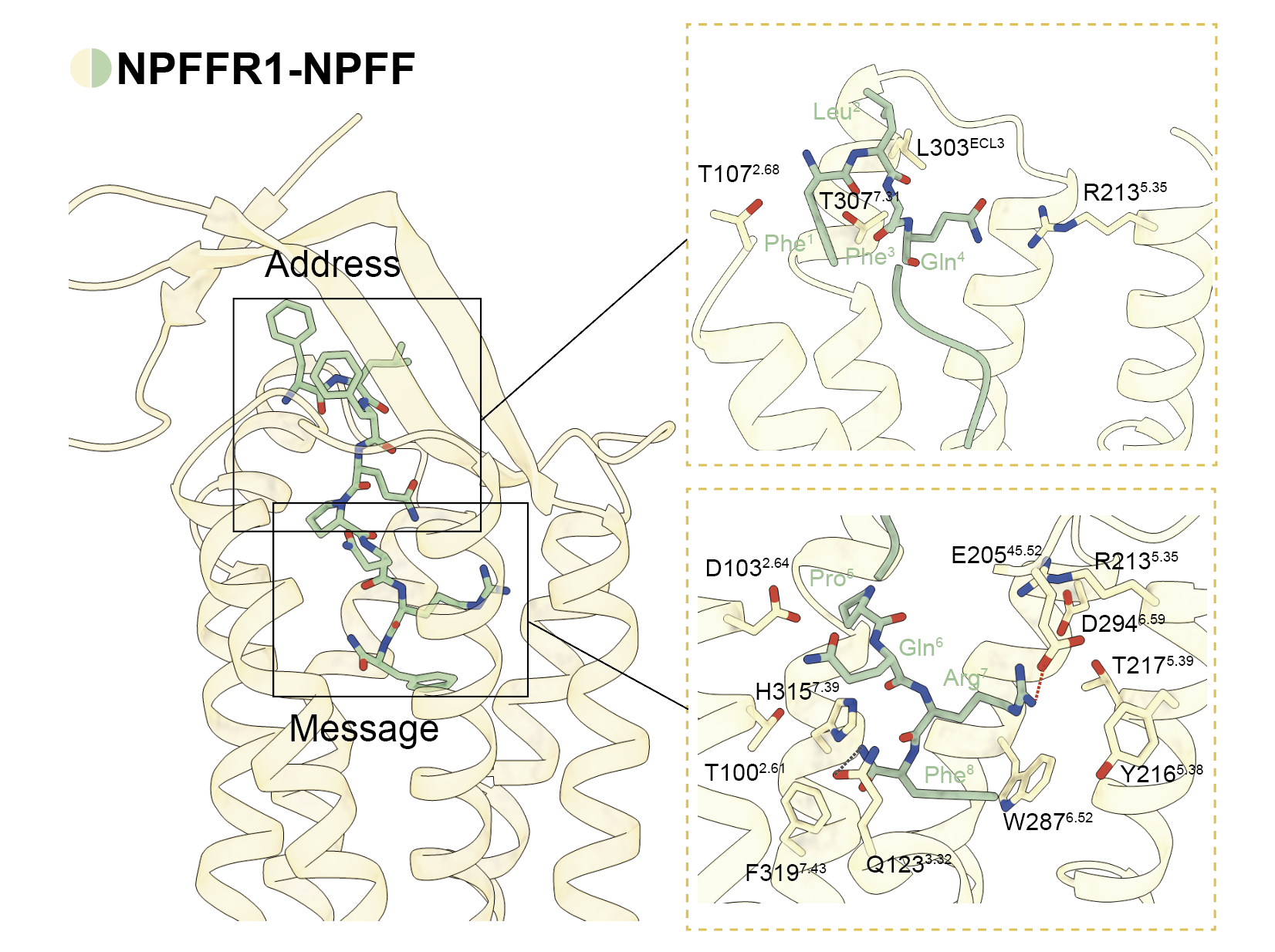


**Fig. S7. Detailed interactions between NPFF and NPFFR1.** The NPFFR1 residues involved in recognition are colored in yellow sticks, hydrogen bond is shown in grey dashed lines, ionic bonds are shown in red dashed lines. In NPFF, the side chain of Arg7 is slightly displaced away from T217^5.39^ and Y216^5.38^, potentially resulting in a weaker interaction with the receptor compared to that of RFRP-3. Residue Gln^6^ is positioned in a hydrophobic sub-pocket of the orthosteric binding site, stabilized by residue D103^2.64^ through hydrophobic interactions (Table S1).


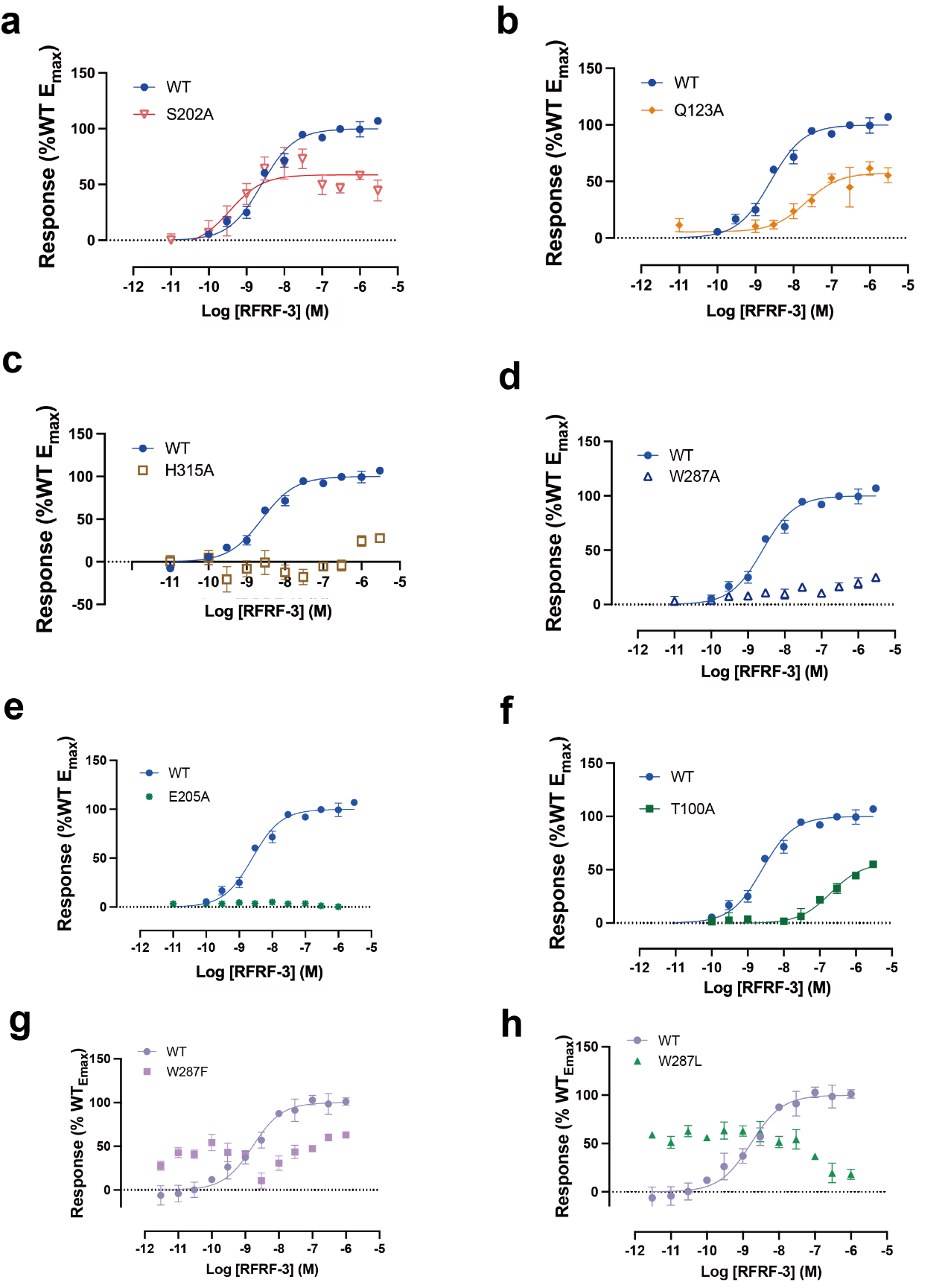


**Fig. S8. Dose-response curves of Gi signaling for wild type (WT) NPFFR1 and mutants activated by RFRP-3 measured by GloSensor assay.** Data shown are mean ± S.E.M. of three independent experiments (n = 3).


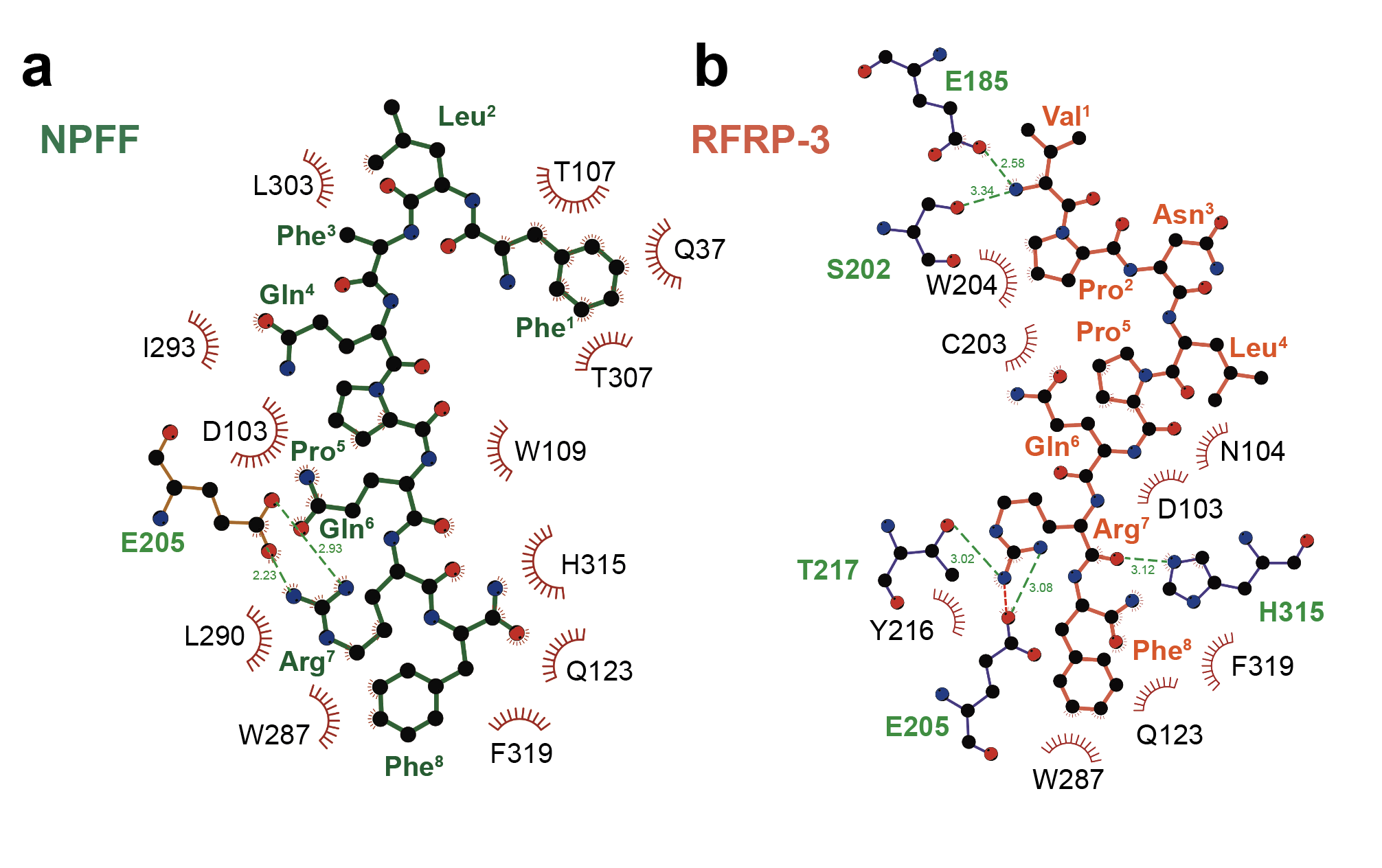


**Fig. S9. Contact maps of NPFF (a) and RFRP-3 (b) in NPFFR1 orthosteric binding pocket.** Hydrophobic residues and interactions are colored in brick red, hydrogen bonds are colored in green dashed lines, polar interactions are colored in red dashed line. The distance between atoms forming hydrogen bonds are labeled in green. Figures made by LigPlot (Wallace, Laskowski et al. 1995).


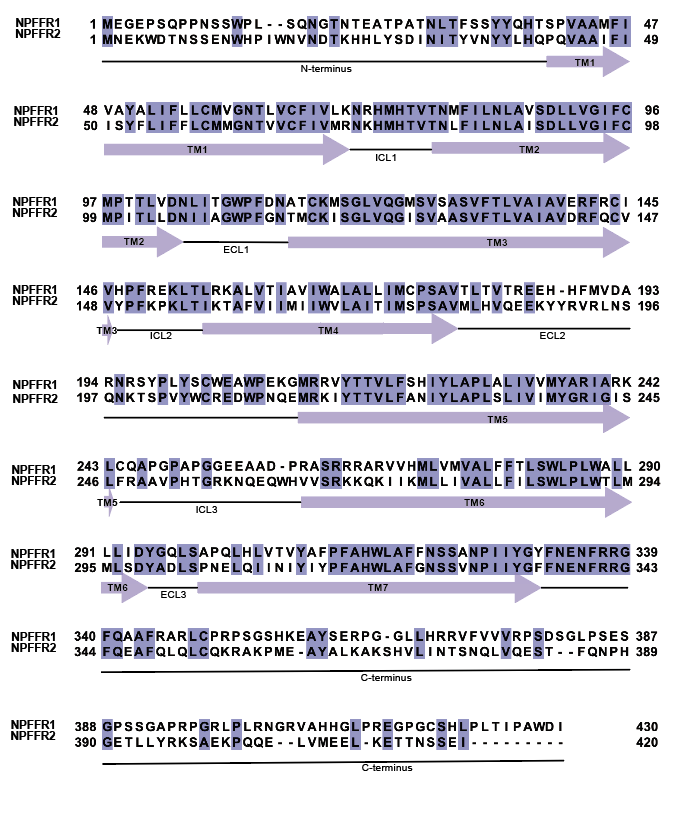


**Fig. S10. Sequence alignment of NPFFR1 and NPFFR2.**The sequence alignment was generated with Jalview and depicts the N-/C-terminus, transmembrane helices (TMs), extracellular loops (ECLs), and intracellular loops (ICLs).


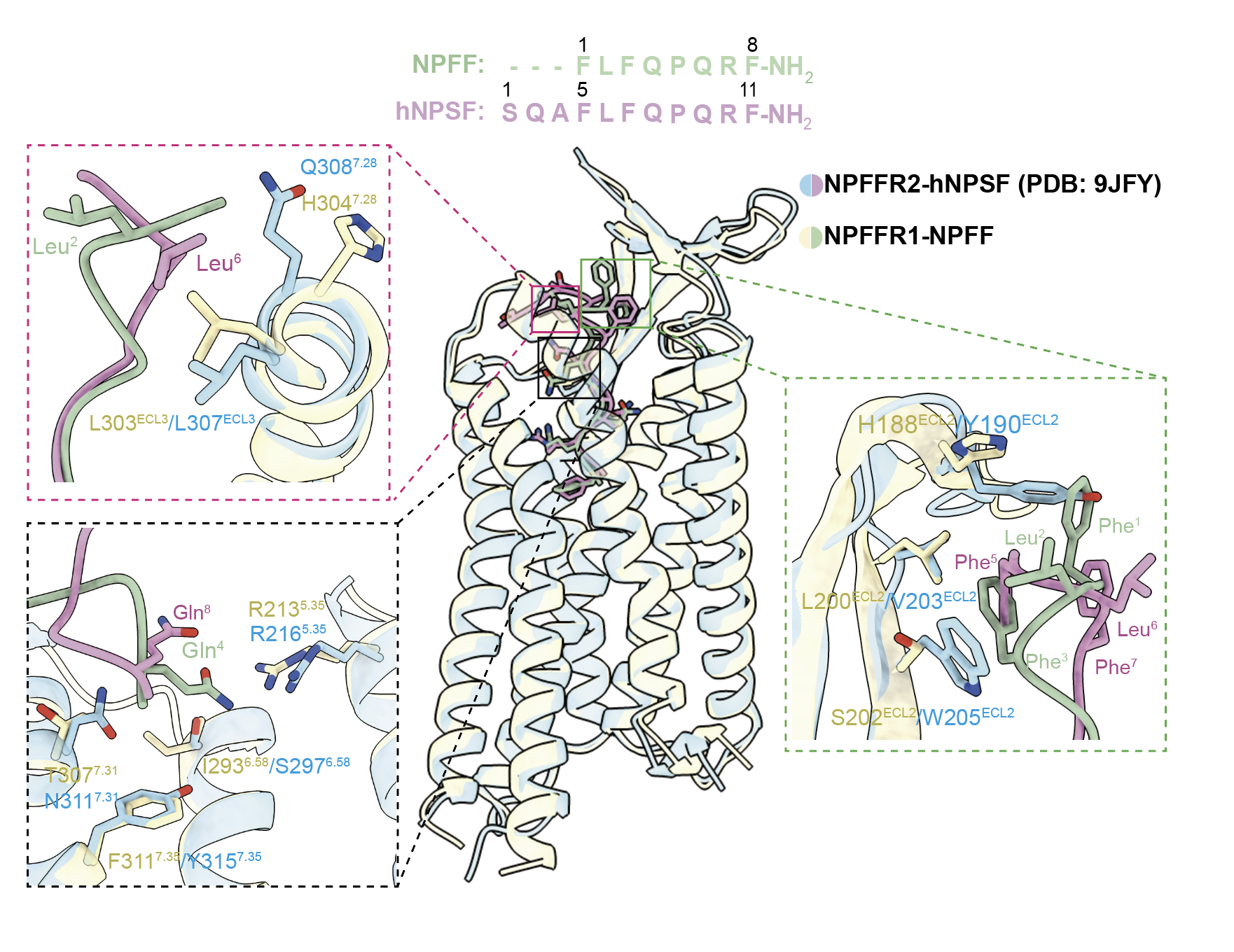


**Fig. S11. Overall comparison of NPFF-NPFFR1 and hNPSF-NPFFR2 (PDB: 9JFY) structures and zoomed view of ligands “address” binding pockets.** NPFFR1 (yellow); NPFFR2 (blue); NPFF (green); hNPSF (pink). NPFF and hNPSF sequences are shown at the top in green and pink respectively, and the residue numbering from the N-termini to the C-termini is shown above the sequence in black. Specifically, Gln^8^ in hNPSF forms water-mediated polar interactions with NPFFR2 residues N311^7.31^, Y315^7.35^, S297^6.58^, and R216^5.35^. However, in NPFFR1, these polar residues — except for R^5.35^ — are replaced by non-polar residues such as I293^6.58^ and F311^7.35^, or by polar residues with smaller side chains like T307^7.31^. In the hNPSF-bound NPFFR2 structure, Leu^6^ engages in interactions with L307^ECL2^ and Q308^7.28^, but these interactions are disrupted in NPFFR1 due to a slight outward shift of TM7 and associated conformation of NPFF, which increases the distance between Leu^2^ and the corresponding residues. Additionally, Phe^5^ and Phe^7^ of hNPSF are stabilized by a hydrophobic interaction network involving Y190^ECL2^, V203^ECL2^, and W205^ECL2^ in NPFFR2. In contrast, NPFFR1 features polar residues at these positions, such as H188^ECL2^ and S202^ECL2^, which may prevent similar hydrophobic interactions with NPFF.


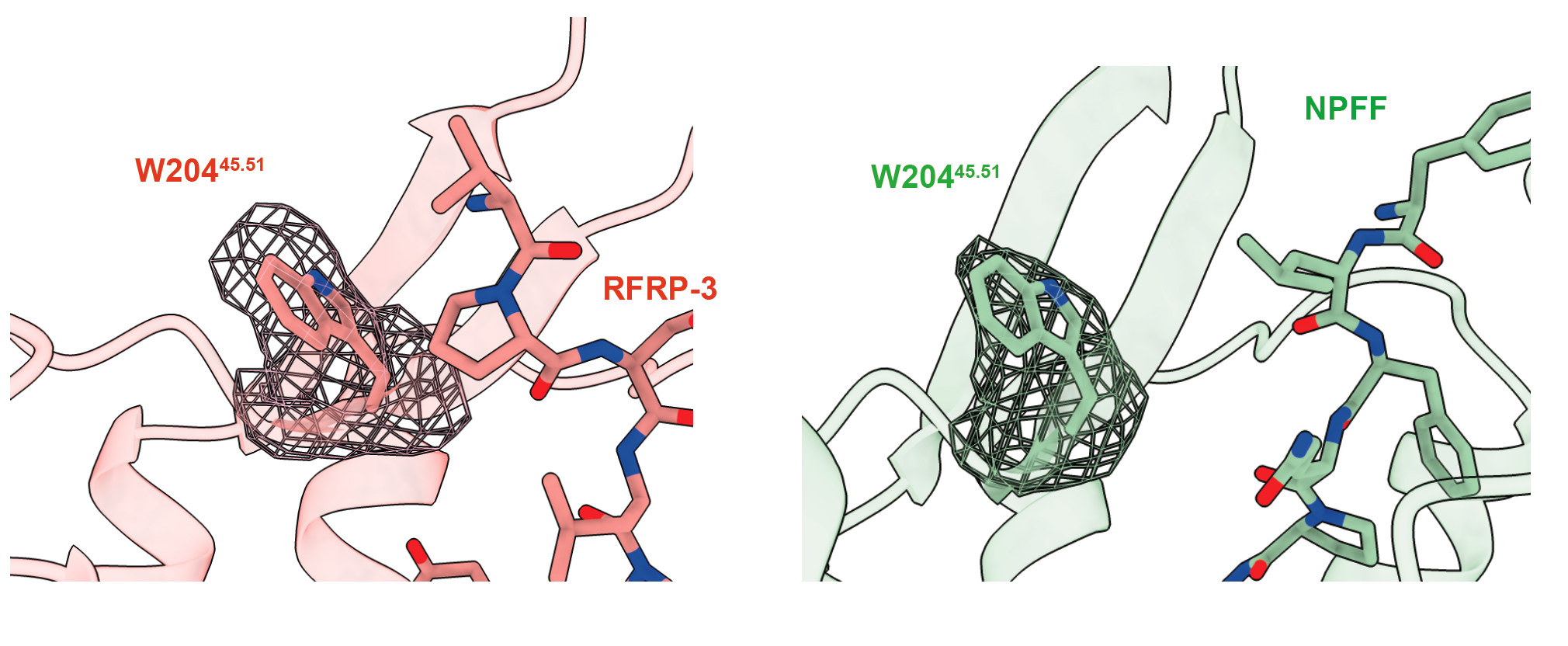


**Fig. S12. Cryo-EM density map of residue W204^45.51^.** RFRP-3-NPFFR1 complex is shown in pink, NPFF-NPFFR1 complex is shown in green.


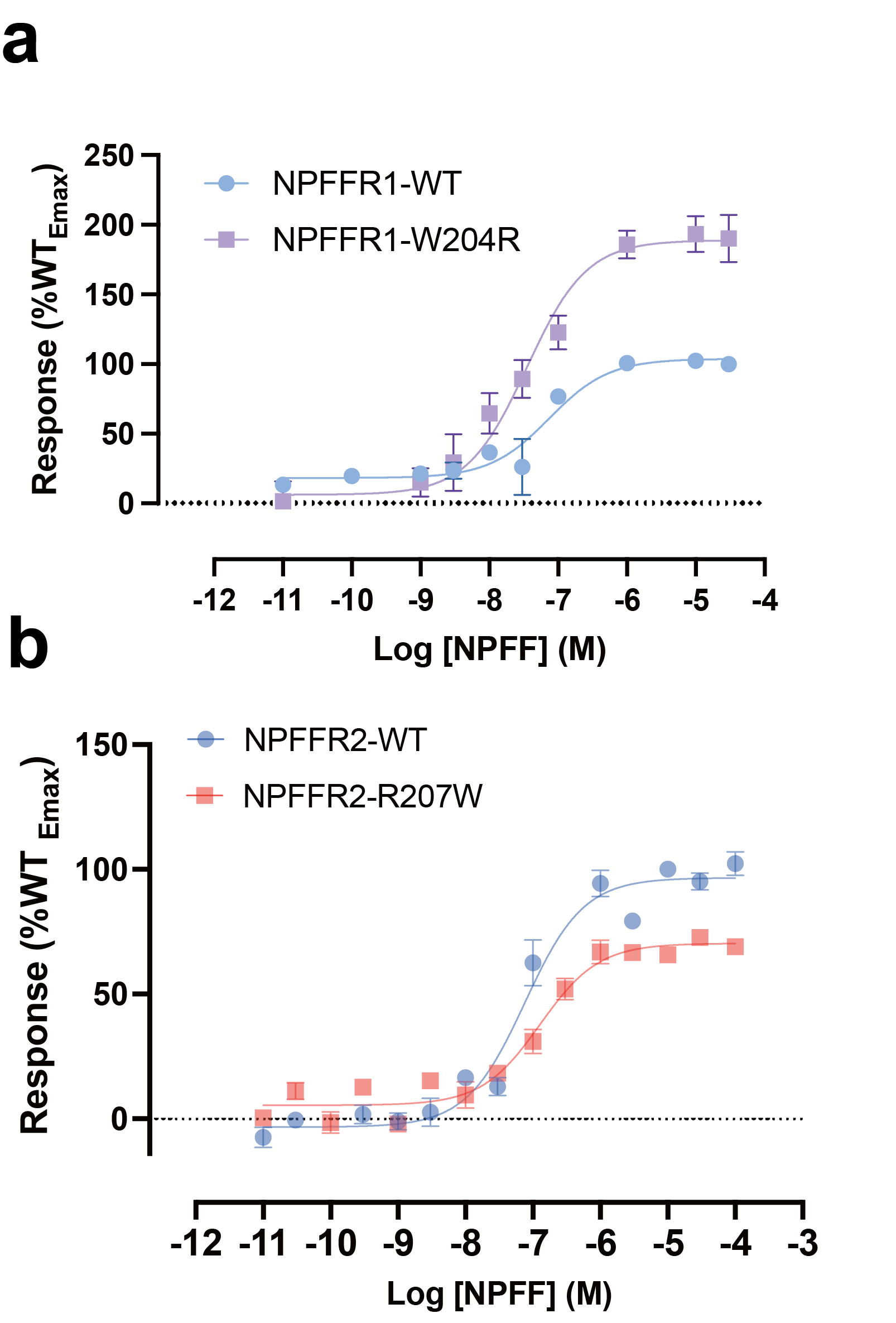


**Fig. S13. Dose-response curves of Gi signaling** for WT-NPFFR1 and its W204R mutant (**a**) / WT-NPFFR2 and its R207W mutant (**b**) activated by NPFF measured by GloSensor assay. Data shown are mean ± S.E.M. of three independent experiments (n = 3).


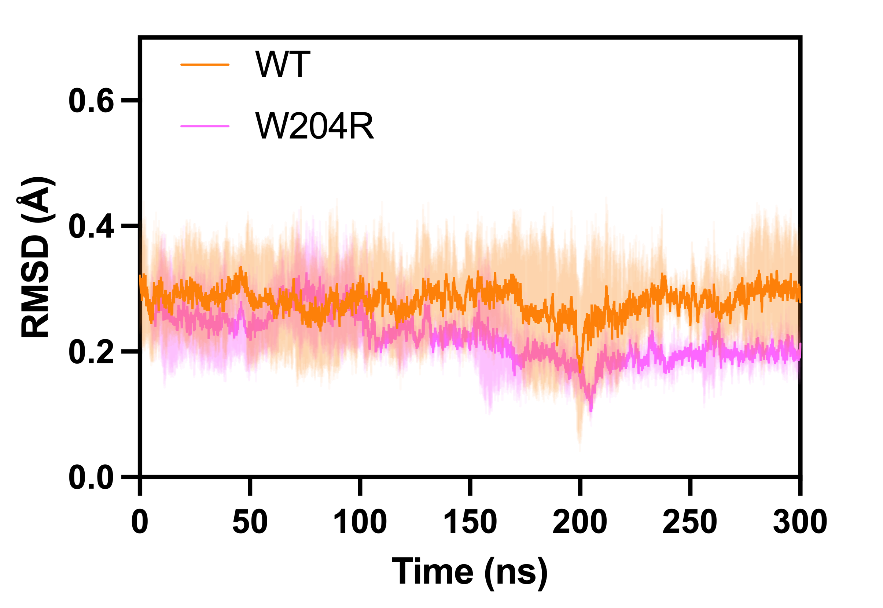


**Fig. S14. MD simulation of the ECL2 region (residues T181-K210) between the wild-type NPFFR1 (WT) and the W204^45.51^R mutant**, which corresponds to the equivalent residue in NPFFR2 (n = 3).


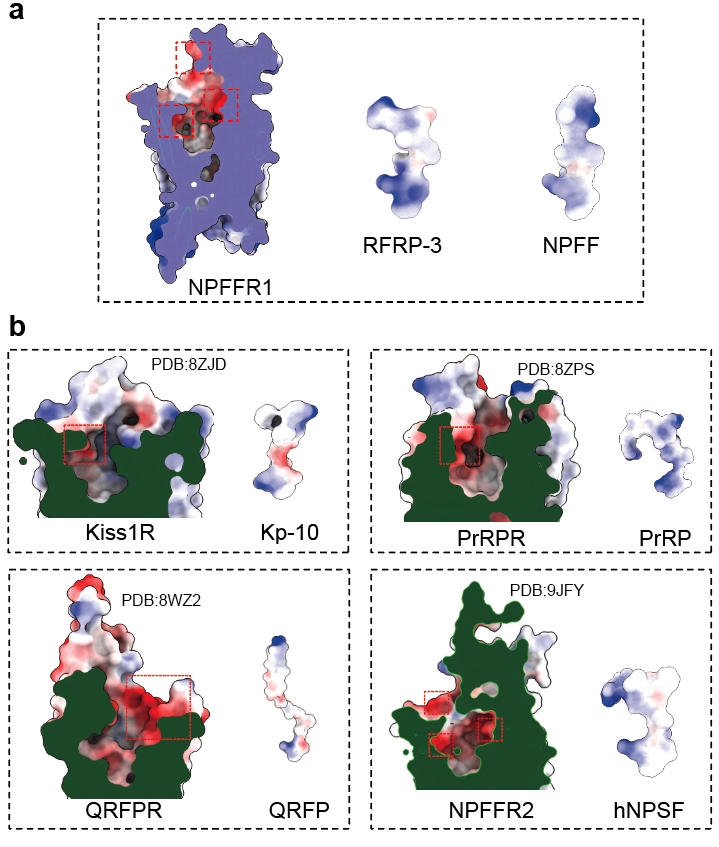


**Fig. S15. Electrostatic surface representation of RF-amide family peptides and receptors.** The figure shows the electrostatic surface of NPFFR1 and its endogenous peptides (**a**), and other RF-amide receptors and their peptides (**b**). Negatively charged regions are highlighted with red dashed rectangles. The electrostatic surfaces of RF-amide peptides are mostly positively charged.


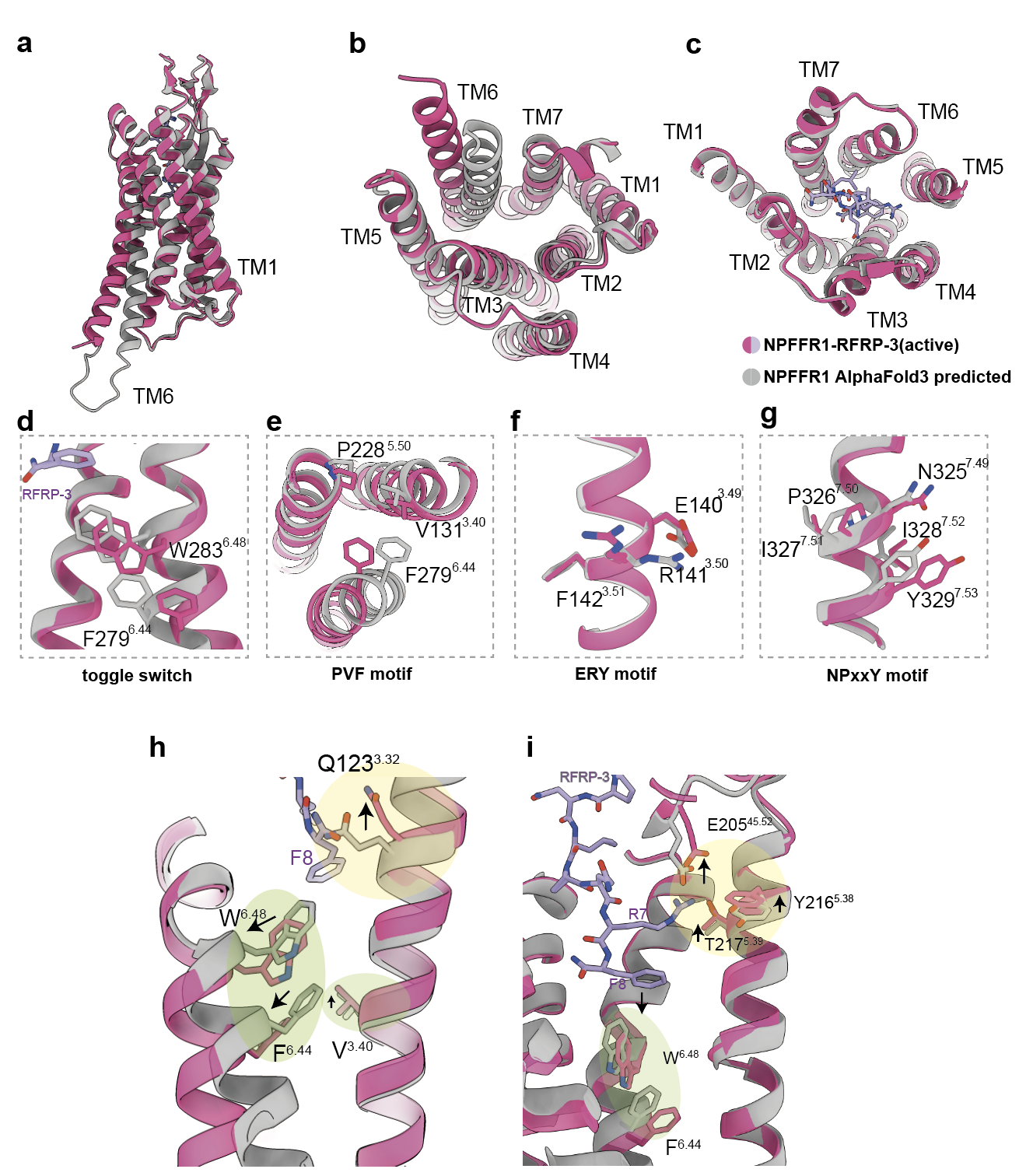


**Fig. S16.** **Activation mechanism of NPFFR1. a.** Superposition of RFRP-3 (purple) -bound NPFFR1 (rose pink, active-state structure) with inactive AlphaFold3 predicted NPFFR1 (grey, inactive-state structure). **b-c.** Comparison of extracellular **(c)** and cytoplasmic **(b)** views of active and inactive states. TM6 exhibits an outward movement of approximately 9 Å. This outward shift is accompanied by inward movements of TM5 and TM7, adopting a fully active conformation **d-g.** Conformational changes in conserved motifs, including the toggle switch, P^5.50^V^3.40^F^6.44^, E^3.49^R^3.50^F^3.51^ (ERY) and N^7.49^P^7.50^xxY^7.53^. **h-i.** Activation of NPFFR1 triggered by ligand, the conserved motifs are shaded in green circle, and receptor residues directly interacting with the ligand are shaded in yellow circle, movements at these residues (from inactive to active) are shown in black arrowheads. Specifically, we found that upon ligand binding, the Q123^3.32^ residue of the receptor shifts upward, causing the upper portion of TM3 to extend in the same direction. This upward movement also promotes an outward and upward displacement of V131^3.40^ by approximately 1.4 Å, weakening the interaction between V131^3.40^ and F279^6.44^ and thereby allowing greater flexibility in the outward movement of F279^6.44^**(h)**. Additionally, residues E205^45.52^, Y216^5.38^, and T217^5.39^ of the receptor facilitate an upward movement of the ligand’s C-terminal Arg^7^ side chain. This motion disrupts the intramolecular cation–π interaction between Arg^7^ and the neighboring Phe^8^, enabling enhanced interaction between the phenyl ring of Phe^8^ and the receptor residue W283^6.48^, thereby promoting receptor activation **(i)**.

**Table S1. Expression level and assay result of WT-NPFFR1 and mutants on NPFF/RFRP-3 induced cAMP accumulation assay.**

| **RFRP-3** | | | **Expression Level ± SEM(%WT)** |
| --- | --- | --- | --- |
| **Gi signaling** | | |  |
|  | **pEC50 ± SEM** | **Emax ± SEM(%WT)** |  |
| **WT** | 8.7 ± 0.05 | 100 | 100 |
| **H315A** | ND | ND | 98 ± 18.09 ^ns^ |
| **Q123A** | 8.2 ± 0.04 ** | 45 ± 7.14 **** | 88 ± 12.74 ^ns^ |
| **D103A** | ND | ND | 106 ± 14.86 ^ns^ |
| **Y216A** | ND | ND | 72.35 ± 5.55 ^ns^ |
| **T217A** | ND | ND | 76.60 ± 7.724 ^ns^ |
| **W204A** | ND | ND | 33.06 ± 11.06 ** |
| **E185A** | ND | ND | 34.94 ± 6.98 ** |
| **T100A** | 6.7 ± 0.4 **** | 54.13 ± 3.06 **** | 126.4 ± 13.57 ^ns^ |
| **S202A** | 9.7 ± 0.04 **** | 84.66 ± 2.01 * | 85.63 ± 7.97 ^ns^ |
| **W287A** | ND | ND | 111.4 ± 8.57 ^ns^ |
| **W287F** | ND | ND | 100.3 ± 8.49 ^ns^ |
| **W287L** | ND | ND | 121.3. ± 10.01 ^ns^ |
|  |  |  |  |
|  |  |  |  |
| **NPFF** | | | **Expression Level ± SEM(%WT)** |
| **Gi signaling** | | |  |
|  | **pEC50 ± SEM** | **Emax ± SEM(%WT)** |  |
| **WT** | 7.4 ± 0.04 | 100 | 100 |
| **D103A** | 6.9 ± 0.13 * | 110 ± 2.53 ^ns^ | 106 ± 14.86 ^ns^ |
| **W204R** | 7.3 ± 0.09 ^ns^ | 198 ± 0.13 **** | 124.2 ± 14.51 ^ns^ |
| **S202W** | 7.1 ± 0.09 ^ns^ | 95 ± 0.05 ^ns^ | 82.48 ± 7.94 ^ns^ |
| **H188Y** | 7.4 ± 0.28 ^ns^ | 93 ± 0.1 ^ns^ | 78.66 ± 9.89 ^ns^ |

The data are presented as the mean ± S.E.M of three independent experiments performed in triplicates. ND indicates no detectable signal. ns, no significance, P > 0.05, *P < 0.0332, **P < 0.0002, and ***P < 0.0001 by one-way ANOVA followed by Dunnett’s multiple comparisons test with WT-NPFFR1.

**Table S2. Cryo-EM data collection, refinement and validation statistics of two NPFFR1-Gi complexes.**

|  | NPFF-NPFFR1-G_i_ | RFRP-3-NPFFR1-G_i_ |
| --- | --- | --- |
| Magnification | 105,000 | 105,000 |
| Voltage (kv) | 300 | 300 |
| Electron exposure (e-/Å^2^) | 60 | 60 |
| Defocus range (µm) | -0.7~-2.2 | -0.7~-2.2 |
| images | 11,340 | 9,306 |
| Pixel size (Å) | 1.04 | 1.04 |
| Symmetry imposed | C1 | C1 |
| Final particles | 270,038 | 140,756 |
| Map resolution | 3.19 | 3.02 |
| FSC threshold | 0.143 | 0.143 |
| R.M.S. deviations |  |  |
| Bond lengths (Å) | 0.006 | 0.005 |
| Bond angles (˚) | 0.689 | 0.722 |
| Validation |  |  |
| MolProbity score | 1.73 | 2.00 |
| Clash score | 9.17 | 8.56 |
| Ramachandran plot |  |  |
| Favored (%) | 96.36 | 96.01 |
| Allowed (%) | 3.55 | 3.99 |
| Disallowed (%) | 0.09 | 0.00 |

**Table S3. Expression level and assay result of WT-NPFFR2 and mutants on NPFF/RFRP-3 induced cAMP accumulation assay.**

| **NPFF** | | | **Expression Level ± SEM(%WT)** |
| --- | --- | --- | --- |
| **Gi signaling** | | |  |
|  | **pEC50 ± SEM** | **Emax ± SEM(%WT)** |  |
| **WT** | 7.8 ± 0.37 | 100 | 100 |
| **R207W** | 7.6 ± 0.36 ^ns^ | 62.57 ± 4.16 **** | 110.7 ± 19.76 ^ns^ |

| **RPRF-3** | | | **Expression Level ± SEM(%WT)** |
| --- | --- | --- | --- |
| **Gi signaling** | | |  |
|  | **pEC50 ± SEM** | **Emax ± SEM(%WT)** |  |
| **WT** | 7.8 ± 0.37 | 100 | 100 |
| **T220A** | ND | ND | 112.7 ± 19.22 ^ns^ |

The data are presented as the mean ± S.E.M of three independent experiments performed in triplicates. ND indicates no detectable signal. ns, no significance, P > 0.05, *P < 0.0332, **P < 0.0002, and ***P < 0.0001 by one-way ANOVA followed by Dunnett’s multiple comparisons test with WT-NPFFR1.

**References**

Chen, V. B., W. B. Arendall, 3rd, J. J. Headd, D. A. Keedy, R. M. Immormino, G. J. Kapral, L. W. Murray, J. S. Richardson and D. C. Richardson (2010). "MolProbity: all-atom structure validation for macromolecular crystallography." Acta Crystallogr D Biol Crystallogr **66**(Pt 1): 12-21.

Chun, E., A. A. Thompson, W. Liu, C. B. Roth, M. T. Griffith, V. Katritch, J. Kunken, F. Xu, V. Cherezov, M. A. Hanson and R. C. Stevens (2012). "Fusion partner toolchest for the stabilization and crystallization of G protein-coupled receptors." Structure **20**(6): 967-976.

Darden, T., D. York and L. Pedersen (1993). "Particle mesh Ewald: An N⋅log(N) method for Ewald sums in large systems." The Journal of Chemical Physics **98**(12): 10089-10092.

Emsley, P., B. Lohkamp, W. G. Scott and K. Cowtan (2010). "Features and development of Coot." Acta Crystallogr D Biol Crystallogr **66**(Pt 4): 486-501.

Hess, B. (2008). "P-LINCS:  A Parallel Linear Constraint Solver for Molecular Simulation." J Chem Theory Comput **4**(1): 116-122.

Hua, T., X. Li, L. Wu, C. Iliopoulos-Tsoutsouvas, Y. Wang, M. Wu, L. Shen, C. A. Brust, S. P. Nikas, F. Song, X. Song, S. Yuan, Q. Sun, Y. Wu, S. Jiang, T. W. Grim, O. Benchama, E. L. Stahl, N. Zvonok, S. Zhao, L. M. Bohn, A. Makriyannis and Z. J. Liu (2020). "Activation and Signaling Mechanism Revealed by Cannabinoid Receptor-G(i) Complex Structures." Cell **180**(4): 655-665.e618.

Huang, J. and A. D. MacKerell, Jr. (2013). "CHARMM36 all-atom additive protein force field: validation based on comparison to NMR data." J Comput Chem **34**(25): 2135-2145.

Huang, J., S. Rauscher, G. Nawrocki, T. Ran, M. Feig, B. L. de Groot, H. Grubmüller and A. D. MacKerell, Jr. (2017). "CHARMM36m: an improved force field for folded and intrinsically disordered proteins." Nat Methods **14**(1): 71-73.

Liang, Y. L., P. Zhao, C. Draper-Joyce, J. A. Baltos, A. Glukhova, T. T. Truong, L. T. May, A. Christopoulos, D. Wootten, P. M. Sexton and S. G. B. Furness (2018). "Dominant Negative G Proteins Enhance Formation and Purification of Agonist-GPCR-G Protein Complexes for Structure Determination." ACS Pharmacol Transl Sci **1**(1): 12-20.

Lomize, M. A., I. D. Pogozheva, H. Joo, H. I. Mosberg and A. L. Lomize (2012). "OPM database and PPM web server: resources for positioning of proteins in membranes." Nucleic Acids Res **40**(Database issue): D370-376.

McCoy, A. J., R. W. Grosse-Kunstleve, P. D. Adams, M. D. Winn, L. C. Storoni and R. J. Read (2007). "Phaser crystallographic software." J Appl Crystallogr **40**(Pt 4): 658-674.

Pettersen, E. F., T. D. Goddard, C. C. Huang, G. S. Couch, D. M. Greenblatt, E. C. Meng and T. E. Ferrin (2004). "UCSF Chimera--a visualization system for exploratory research and analysis." J Comput Chem **25**(13): 1605-1612.

Punjani, A., J. L. Rubinstein, D. J. Fleet and M. A. Brubaker (2017). "cryoSPARC: algorithms for rapid unsupervised cryo-EM structure determination." Nat Methods **14**(3): 290-296.

Ranganathan, A., R. O. Dror and J. Carlsson (2014). "Insights into the role of Asp79(2.50) in β2 adrenergic receptor activation from molecular dynamics simulations." Biochemistry **53**(46): 7283-7296.

Sanchez-Garcia, R., J. Gomez-Blanco, A. Cuervo, J. M. Carazo, C. O. S. Sorzano and J. Vargas (2021). "DeepEMhancer: a deep learning solution for cryo-EM volume post-processing." Commun Biol **4**(1): 874.

Sastry, G. M., M. Adzhigirey, T. Day, R. Annabhimoju and W. Sherman (2013). "Protein and ligand preparation: parameters, protocols, and influence on virtual screening enrichments." J Comput Aided Mol Des **27**(3): 221-234.

Vanommeslaeghe, K. and A. D. MacKerell, Jr. (2012). "Automation of the CHARMM General Force Field (CGenFF) I: bond perception and atom typing." J Chem Inf Model **52**(12): 3144-3154.

Wallace, A. C., R. A. Laskowski and J. M. Thornton (1995). "LIGPLOT: a program to generate schematic diagrams of protein-ligand interactions." Protein Eng **8**(2): 127-134.

Wu, E. L., X. Cheng, S. Jo, H. Rui, K. C. Song, E. M. Dávila-Contreras, Y. Qi, J. Lee, V. Monje-Galvan, R. M. Venable, J. B. Klauda and W. Im (2014). "CHARMM-GUI Membrane Builder toward realistic biological membrane simulations." J Comput Chem **35**(27): 1997-2004.

Yue, Y., L. Liu, L.-J. Wu, Y. Wu, L. Wang, F. Li, J. Liu, G.-W. Han, B. Chen, X. Lin, R. L. Brouillette, É. Breault, J.-M. Longpré, S. Shi, H. Lei, P. Sarret, R. C. Stevens, M. A. Hanson and F. Xu (2022). "Structural insight into apelin receptor-G protein stoichiometry." Nature Structural & Molecular Biology **29**(7): 688-697.

Zhuang, Y., Y. Wang, B. He, X. He, X. E. Zhou, S. Guo, Q. Rao, J. Yang, J. Liu, Q. Zhou, X. Wang, M. Liu, W. Liu, X. Jiang, D. Yang, H. Jiang, J. Shen, K. Melcher, H. Chen, Y. Jiang, X. Cheng, M. W. Wang, X. Xie and H. E. Xu (2022). "Molecular recognition of morphine and fentanyl by the human μ-opioid receptor." Cell **185**(23): 4361-4375.e4319.
